# Serum Insights: Leveraging the Power of miRNA Profiling as an Early Diagnostic Tool for Non-Small Cell Lung Cancer

**DOI:** 10.3390/cancers15204910

**Published:** 2023-10-10

**Authors:** Radoslaw Charkiewicz, Anetta Sulewska, Robert Mroz, Alicja Charkiewicz, Wojciech Naumnik, Marcin Kraska, Attila Gyenesei, Bence Galik, Sini Junttila, Borys Miskiewicz, Rafal Stec, Piotr Karabowicz, Magdalena Zawada, Wojciech Miltyk, Jacek Niklinski

**Affiliations:** 1 Center of Experimental Medicine, Medical University of Bialystok, 15-369 Bialystok, Poland; 2 Department of Clinical Molecular Biology, Medical University of Bialystok, 15-269 Bialystok, Poland; anetta.sulewska@umb.edu.pl (A.S.); marcin.kraska92@gmail.com (M.K.); 3 2nd Department of Lung Diseases and Tuberculosis, Medical University of Bialystok, 15-540 Bialystok, Poland; robert.mroz@umb.edu.pl; 4 Department of Analysis and Bioanalysis of Medicines, Medical University of Bialystok, 15-089 Bialystok, Poland; alicja.charkiewicz@umb.edu.pl (A.C.); wojciech.miltyk@umb.edu.pl (W.M.); 5 1st Department of Lung Diseases and Tuberculosis, Medical University of Bialystok, 15-540 Bialystok, Poland; wojciech.naumnik@umb.edu.pl; 6 Department of Medical Pathomorphology, Medical University of Bialystok, 15-269 Bialystok, Poland; 7 Szentagothai Research Center, Genomic and Bioinformatic Core Facility, H-7624 Pecs, Hungary; gyenesei.attila@pte.hu (A.G.); galik.bence@pte.hu (B.G.); 8 Turku Bioscience Centre, University of Turku & Åbo Akademi University, FI-20520 Turku, Finland; simaju@utu.fi; 9 Department of Thoracic Surgery, Medical University of Bialystok, 15-276 Bialystok, Poland; borys.miskiewicz@uskwb.pl; 10 Department of Oncology, Medical University of Warsaw, 02-091 Warsaw, Poland; rafal.stec@uckwum.edu.pl; 11 Biobank, Medical University of Bialystok, 15-269 Bialystok, Poland; piotr.karabowicz@umb.edu.pl; 12 Department of Hematology Diagnostics and Genetics, The University Hospital, 30-688 Krakow, Poland; mzawada@su.krakow.pl

**Keywords:** miRNA, non-small cell lung cancer, liquid biopsy, biomarkers, next-generation sequencing, gradient-boosting decision tree classifier, Shapley Additive exPlanations

## Abstract

**Simple Summary:**

Non-small cell lung cancer (NSCLC) is a prevalent and lethal disease. Circulating cell-free miRNA has the potential to serve as a biomarker for early detection as it reflects cancer characteristics. Through global miRNA profiling in serum samples from NSCLC patients and non-cancerous individuals, we identified 28 upregulated miRNAs in NSCLC and explored their relevance to NSCLC-related pathways. Harnessing an advanced machine-learning algorithm, we successfully developed a robust classifier capable of distinguishing NSCLC from non-cancerous cases. Our findings suggest that serum miRNAs hold promise as a valuable tool for early NSCLC diagnosis and offer valuable insights into NSCLC biology. To solidify these promising results, further validation in diverse patient cohorts is essential.

**Abstract:**

Non-small cell lung cancer is the predominant form of lung cancer and is associated with a poor prognosis. MiRNAs implicated in cancer initiation and progression can be easily detected in liquid biopsy samples and have the potential to serve as non-invasive biomarkers. In this study, we employed next-generation sequencing to globally profile miRNAs in serum samples from 71 early-stage NSCLC patients and 47 non-cancerous pulmonary condition patients. Preliminary analysis of differentially expressed miRNAs revealed 28 upregulated miRNAs in NSCLC compared to the control group. Functional enrichment analyses unveiled their involvement in NSCLC signaling pathways. Subsequently, we developed a gradient-boosting decision tree classifier based on 2588 miRNAs, which demonstrated high accuracy (0.837), sensitivity (0.806), and specificity (0.859) in effectively distinguishing NSCLC from non-cancerous individuals. Shapley Additive exPlanations analysis improved the model metrics by identifying the top 15 miRNAs with the strongest discriminatory value, yielding an AUC of 0.96 ± 0.04, accuracy of 0.896, sensitivity of 0.884, and specificity of 0.903. Our study establishes the potential utility of a non-invasive serum miRNA signature as a supportive tool for early detection of NSCLC while also shedding light on dysregulated miRNAs in NSCLC biology. For enhanced credibility and understanding, further validation in an independent cohort of patients is warranted.

## 1. Introduction

Lung cancer (LC) is a major contributor to cancer-related mortality globally, and its prognosis primarily depends on the stage of diagnosis. Unfortunately, a large proportion of cases are diagnosed at locally advanced or advanced stages, when curative treatment is not feasible. Consequently, enhancing the accuracy of early detection of LC is critical to improve treatment outcomes, reduce mortality, and minimize healthcare costs and adverse events associated with systemic therapies [1,2].

Non-small cell lung cancer (NSCLC) constitutes approximately 85% of all LC cases [3]. Current methods for NSCLC screening and diagnosis are often invasive, expensive, and have low sensitivity and specificity [4]. For instance, chest X-rays are not capable of distinguishing cancer from other conditions; sputum cytology, bronchoscopy, needle biopsy, and thoracentesis are invasive procedures that may result in complications and discomfort; and low-dose computed tomography (LDCT) has several limitations, including high false-positive rates, radiation exposure, and overdiagnosis [5,6].

Histopathology serves as the conventional diagnostic modality for NSCLC; nonetheless, it exhibits inherent limitations. Insufficient tissue acquisition or distorted tissue architecture in small biopsies and cytologic specimens can impede early detection. Moreover, the reliance on the pathologist’s expertise and experience introduces potential variability and subjectivity in interpretation [7,8].

Biomarker testing represents a step towards personalized medicine, which aims to improve diagnosis and provide tailored treatments based on individual patient characteristics (e.g., EGFR, ALK, and PD-L1). However, biomarker testing also presents challenges, such as requiring adequate and representative tissue or blood samples for analysis, variation between and complexity of testing methods across different laboratories or platforms, and lack of standardization and validation of some biomarkers across different populations or settings [7,8]. Thus, new methods and biomarkers are urgently needed for early, non-invasive diagnosis of NSCLC to improve the accuracy, sensitivity, and specificity of NSCLC diagnosis and offer personalized treatment options.

MicroRNA (miRNA) testing is an emerging field advancing personalized medicine, aiming to improve diagnosis and provide tailored treatments based on individual patient characteristics [9]. MicroRNAs are small non-coding RNAs that regulate gene expression at the post-transcriptional level [10]. They are implicated in various biological processes and diseases, including LC development and progression, as evidenced by in vitro studies [11,12]. MiRNAs are detectable in various body fluids, such as blood, urine, saliva, and cerebrospinal fluid. Circulating cell-free miRNAs (cf-miRNAs) have several advantages as diagnostic biomarkers for NSCLC, including stability, abundance, and specificity. Furthermore, cf-miRNAs can predict drug response by monitoring genetic profiles during treatment, presenting significant potential for personalized therapy [13,14].

In our previous studies (2016–2023), we explored genetic and epigenetic changes in NSCLC. We found hsa-miR-205 and hsa-miR-21 to be promising biomarkers for early NSCLC diagnosis, distinguishing between adenocarcinoma (AC) and squamous cell carcinoma (SCC) subtypes with 88% agreement [15]. Using microarray technology, we developed a 53-gene signature with 93% accuracy in distinguishing between AC and SCC [16]. Employing miRNA-Seq on NSCLC tissue, we crafted a 17-miRNA signature that effectively differentiated NSCLC subtypes (AC vs. SCC) and demonstrated a remarkable area under the curve (AUC) value of 0.994 [17]. Additionally, a 14-lncRNA signature effectively detected NSCLC and provided subtyping information (AUC values: 0.98 ± 0.01 for tumor vs. non-tumor and 0.84 ± 0.09 for subtyping) [18]. 

Expanding upon the foundation established by our prior investigations, the main aim of the present study was to explore the feasibility of serum circulating cell-free microRNAs as non-invasive, cost-effective, and accurate biomarkers for early detection of NSCLC. To accomplish this objective, we have undertaken the following tasks: (a) conducting global miRNA profiling in liquid biopsy samples obtained from early-stage NSCLC patients and non-cancerous patients with pulmonary conditions by employing next-generation sequencing (NGS); (b) identifying the most relevant serum DEmiRNAs to differentiate between NSCLC and non-cancer pulmonary conditions; (c) performing functional analysis of the serum DEmiRNA profile to elucidate potential biological pathways and molecular mechanisms involved in NSCLC development and progression.; and (d) establishing a serum miRNA signature for early detection of NSCLC by using a gradient-boosting decision tree classifier (GBDT) and Shapley Additive exPlanations (SHAP) analysis. 

Our study presents a novel, comprehensive approach to miRNA analysis, setting it apart from other NGS-based miRNA studies as it seamlessly integrates the capabilities of NGS technology with advanced machine learning tools. This synergistic approach provides an in-depth picture of serum miRNA expression profiling, enabling its potential use as a diagnostic tool for NSCLC. The successful realization of this achievement can be attributed to the integration of several critical components, resulting in a robust and insightful methodology and statistical analysis. To begin, we prioritized the use of rigorously clinically characterized groups. Through meticulous participant selection and thorough evaluation, we ensured that the individuals included in our study met the specific research criteria. This rigorous approach aimed to enhance the reliability and validity of our findings. Furthermore, we implemented stringent controls at every stage of the analytical processes. By closely monitoring and regulating each step, we effectively mitigated potential biases and errors, reinforcing the reliability and robustness of our results. In our analysis, we adopted a holistic approach, thoroughly examining the entire repertoire of microRNAs present in the blood serum. By harnessing the power of NGS technology, we conducted a scrutinized analysis to identify circulating miRNAs, including both established and potentially novel ones. To develop a highly accurate serum miRNA signature for the early stage of NSCLC detection, we employed advanced machine learning techniques. Specifically, we utilized a gradient-boosting decision tree classifier and applied Shapley Additive exPlanations (SHAP) analysis. Our research has the potential to be translated into practical applications, such as the development of diagnostic tools for early diagnosis of NSCLC in blood serum. This aspect emphasizes the translational nature of our work, bridging the gap between scientific discoveries and their utilization in clinical practice.

## 2. Materials and Methods

This study was conducted within the framework of the Polish project titled “Development of Personalized Diagnostic of Malignant Tumors based on tumor heterogeneity and integrated genomic, transcriptomic, metabolomic, and imaging PET/MRI analysis. Getting Ready for Individualized Therapy”. Prior to sample collection and clinicopathological data processing, written informed consent was obtained from all participants. The study protocol was reviewed and approved by the Bioethics Committee of the Medical University of Bialystok, with ethical approval code R-I-002/357/2014.

### 2.1. Patients and Samples

Serum blood samples were obtained from a total of 118 individuals, comprising 71 early-stage NSCLC patients and 47 non-cancer patients (21 with chronic obstructive pulmonary disease (COPD) and 26 without COPD) who were recruited from the 1st and 2nd Departments of Pulmonary Diseases and Tuberculosis of the Medical University of Bialystok. It is worth noting that none of the NSCLC patients had previously received chemo- or radiotherapy. The NSCLC group comprised 71 patients, including 31 female and 40 male participants, with a mean age of 66 years. The histologic subtypes of NSCLC were squamous cell carcinoma (SCC, *n* = 36), adenocarcinoma (AC, *n* = 32), large cell carcinoma (LCC, *n* = 2), and NSCLC not otherwise specified (NSCLC-NOS, *n* = 1). The disease stages were distributed as follows: 20 patients in stage IA, 19 in stage IB, 13 in stage IIA, 9 in stage IIB, and 10 in stage IIIA. A total of 66 patients were smokers, and 5 were never smokers. The control group consisted of 47 individuals, or 17 women and 30 men, with a mean age of 64 years. The control group participants were either diagnosed with chronic obstructive pulmonary disease (COPD, *n* = 21) or did not have COPD (noCOPD, *n* = 26). The No-COPD group comprised patients diagnosed with various pulmonary non-neoplastic conditions, including emphysema, bronchitis, pneumonia, fibroma, metabolically active proliferative process, sarcoidosis, chronic cough, and lower respiratory symptoms. All participants in the control group had a history of smoking. The detailed characteristics of the patients can be found in Table 1.

In our study, the inclusion of both COPD samples and non-COPD samples in the control group was driven by the objective of conducting a comprehensive evaluation of miRNA profiles in early-stage NSCLC patients, comparing them to a diverse population of smokers with non-cancerous lung diseases. However, it is important to acknowledge that the control group was limited in size due to various challenges. The availability of samples was constrained by the relatively low number of patients who had the pulmonary non-neoplastic conditions under investigation at the clinics from which we collected the material. Additionally, the willingness of eligible patients to participate in the study also contributed to the constraint in the sample size. Despite these constraints, it is worth noting that the sample size of our study group, consisting of 47 individuals, exceeded those of control groups in other published works that utilized serum miRNA profiling using NGS [19,20].

**Figure 1:**
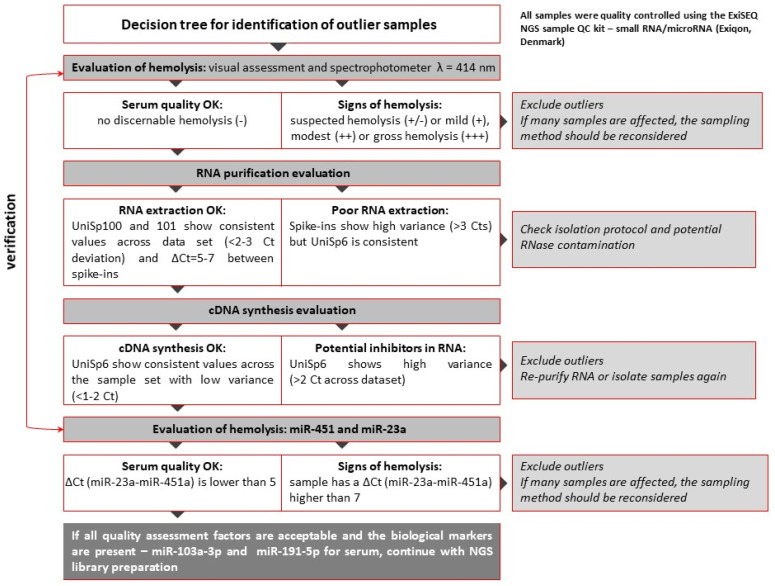
Decision tree for identification of outlier samples.

Aseptic collection of 9 mL of whole blood was performed on participants using S-Monovette Serum Gel tubes (Sarstedt, Nümbrecht, Germany), followed by allowing the samples to naturally clot for 30 min at room temperature. Exclusion criteria involved samples displaying evident indicators of hemolysis, icterus, or lipemia. Subsequently, the clotted blood underwent centrifugation at 2000× *g* for 20 min at zero acceleration and deceleration within a refrigerated centrifuge. Careful transfer of the resulting supernatant was executed into 2 mL Eppendorf tubes. To achieve purified serum, the supernatant was subjected to a secondary centrifugation step at 20,000× *g* for 15 min in a refrigerated centrifuge. Hemolysis in the resulting serum samples was assessed through visual examination and spectrophotometric analysis at a wavelength of 414 nm (Figure 1) [21]. In our study, we focused on hemolysis in erythrocytes as an indicator of sample quality. Erythrocytes are more sensitive to degradation during coagulation compared to leukocytes due to their higher surface area-to-volume ratio, lower osmotic resistance, and fewer protective mechanisms [22,23]. Subsequently, the serum was divided into 0.5 mL aliquots and promptly stored at −80 °C until the subsequent RNA extraction process. 

To ensure precise categorization of cancer patients, confirmation of diagnosis was carried out on formalin-fixed paraffin-embedded (FFPE) tissue specimens. Histopathological assessment adhered to the latest World Health Organization (WHO) classification for lung cancer and the International Multidisciplinary Classification of Lung Adenocarcinoma by the International Association for the Study of Lung Cancer (IASLC), American Thoracic Society (ATS), and European Respiratory Society (ERS). In cases where uncertainty persisted, specimens underwent immunohistochemical staining to assess the expression of thyroid transcription factor-1 (TTF-1), a marker indicative of adenocarcinoma, and p63 protein, an indicator of squamous cell immunophenotype. 

### 2.2. RNA Extraction

**Figure 2:**
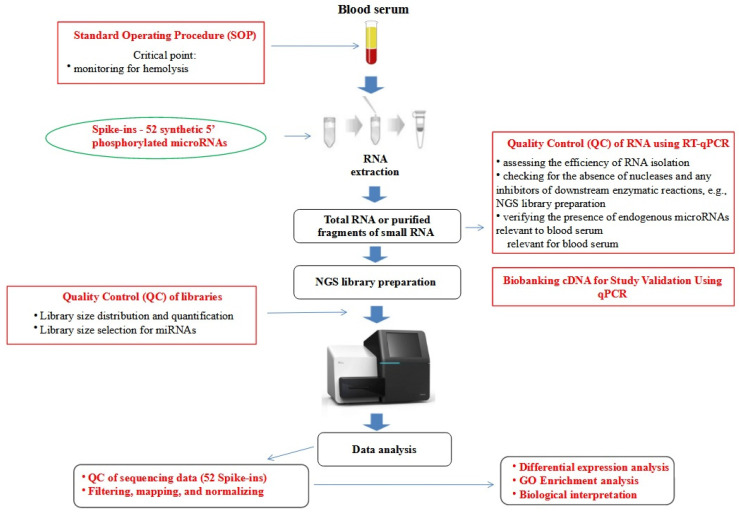
Comprehensive NGS analysis workflow for blood samples: from collection to data analysis.

Prior to RNA extraction, all serum samples were thawed completely on ice, followed by centrifugation at 20,000× *g* for 15 min at 4 °C to eliminate residual cell debris. Total RNA, including the small RNA fraction, was extracted from the serum samples using a modified protocol of the mirVana miRNA Isolation Kit (Invitrogen, Waltham, MA, USA) [24]. To monitor the efficiency of RNA extraction and the presence of nucleases or inhibitors of enzymatic reactions, such as ligation and PCR amplification, a solution of exogenous Spike-ins–52-synthetic 5′ phosphorylated microRNAs (Exiqon, Copenhagen, Denmark) was added to each serum sample prior to isolation (Figure 2). The ExiSEQ NGS sample QC Kit–small RNA/microRNA (Exiqon, Denmark) was used for this purpose. Furthermore, the expression levels of endogenous miRNAs indicative of hemolysis were assessed using this kit to validate the findings from visual and spectrophotometric evaluations (Figure 1 and Figure 2). The quantity of RNA was measured fluorometrically using the Qubit RNA HS Assay Kit (Thermo Scientific, Waltham, MA, USA). Additionally, to determine the concentration of cell-free miRNA (cf-miRNA) in each individual sample, the Qubit microRNA Assay Kit (Thermo Scientific, USA) was utilized. Moreover, small RNA microfluidic chips (Agilent Small RNA kit, Agilent Technologies) were employed, and visual assessment of discernible bands representing the fraction of small RNA was conducted on the capillary gel of electropherograms.

### 2.3. Next Generation Sequencing Analysis (NGS)

**Figure 3:**
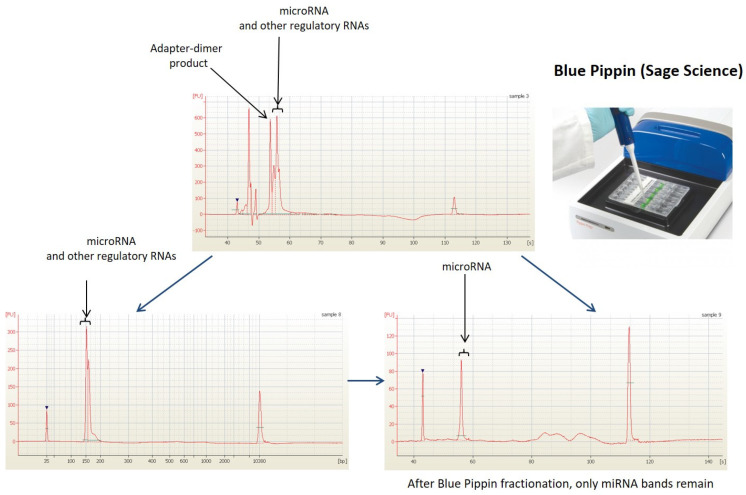
Example of Blue Pippin size fractionation.

To ensure quality control throughout the study, samples were evaluated at each stage prior to the actual next-generation sequencing (NGS) analysis. Outliers were identified and excluded from the study using a decision tree (Figure 1) [21]. The cDNA libraries were prepared using the NEXTflex Small RNA Sequencing Kit v3 (gel-free and low input options) from BioScientific (Phoenix, AZ, USA), which is compatible with Illumina technology. The structure and distribution of each library fraction, representing individual fractions of molecules in the small RNA pool, were assessed using microcapillary electrophoresis with High-Sensitivity DNA chips on the Bioanalyzer 2100 from Agilent Technologies (Santa Clara, CA, USA). Agarose gel electrophoresis with specialized gel cassettes on the Blue Pippin system from Sage Science (Beverly, MA, USA) was employed to select cDNA products of appropriate size corresponding to the miRNA fraction (Figure 3). The concentration of cDNA libraries after fractionation was determined using the KAPA Library Quantification Kit for Illumina Platforms from Roche (San Jose, CA, USA) based on amplification techniques. Sequencing was performed on a HiSeq 4000 instrument from Illumina (San Diego, CA, USA). The detailed decision tree, encompassing hemolysis evaluation, RNA purification, and cDNA synthesis, for the identification of outliers is presented in Figure 1. The workflow for NGS analysis of blood serum samples, from material collection to data analysis, along with quality control points, is depicted in Figure 2.

### 2.4. Bioinformatics and Statistical Analyses

The analyses were performed using the R language version 3.4.1 and environment for statistical computing, with several analyses utilizing the Bioconductor module version 3.5, which provides a range of packages for analyzing biological data. 

Data preprocessing involved the following steps: (a) aligning the transcriptome sequence reads with the reference genome; (b) counting the number of reads for each miRNA; (c) evaluating mapping quality and sample relationships using various methods and visualization techniques (including expression values, correlations, hierarchical clustering, and principal component analysis); (d) excluding samples with low quality or data outliers at this stage; and (e) performing normalization to reduce systematic noise from non-biological sources and improve sample comparability (see more details: Supplementary Materials, Serum miRNA-seq analyses, pages 2 and 3). 

The filtered, differentially expressed (DE) miRNAs were identified based on statistical significance and differences in mean expression levels between sample groups, using fold change (FC) and *p*-values for loose and strict thresholds as filtering parameters (see more details: Supplementary Materials, Serum miRNA-seq analyses, page 30).

Furthermore, we conducted comparative analyses between distinct patient subgroups to elucidate the presence of shared miRNAs across various comparisons, encompassing AC vs. COPD, AC vs. no COPD, SCC vs. COPD, SCC vs. no COPD, AC vs. SCC, NSCLC vs. COPD, NSCLC vs. no COPD, and COPD vs. no COPD. 

Functional enrichment of DEmiRNA targets was performed using GO and KEGG databases, which respectively organize genes into biological processes, molecular functions, and cellular components, and list pathways for biological interactions. The enrichment analysis examined whether miRNA target genes annotated to specific KEGG pathways or GO terms were statistically overrepresented in a given comparison. The mirPath v.3 tool was used for the analysis. 

To construct the diagnostic signature of serum miRNAs, machine learning tools were employed. The binary gradient boosting decision tree classifier was utilized to assess the diagnostic value of all miRNAs. Subsequently, the established models underwent evaluation and analysis using Shapley Additive exPlanations (SHAP) [25] to quantify the significance of specific miRNA values in the model’s predictions. To improve the evaluation scores of the original model, the top 5, 10, 15, 20, and 25 most important variables were selected and tested as inputs. To ensure the robustness of the models, a fivefold stratified cross-validation approach was implemented. Samples were randomly assigned to training and test sets while maintaining balanced classes within each set. This random assignment process was repeated 100 times. For each cross-validation step, accuracy, f1, receiver operating characteristic (ROC) curve, the area under the ROC (AUC), specificity, sensitivity, precision, and negative predictive value (NPV) were computed. The results were reported as mean values accompanied by 95% confidence intervals (CI). The following Python packages were employed to build and examine the models: scikit-learn v1.0.2 [26] for model evaluation, xgboost v1.7.6 [27] for the gradient boosting decision tree classifier, and shap v0.41.0 [25] for the explanatory model analysis.

## 3. Results

### 3.1. Raw Data Preprocessing and Quality Control

The Supplementary Materials (Serum miRNA-seq analysis, pages 2–29) provide extensive information about the analysis workflow (Figure S1), sample description (Table S1), and data preprocessing and quality control steps. Preprocessing steps included alignment metrics for spike-in reads (Table S2) and genomic reads (Table S3), as well as histograms representing the distribution of reads in feature counting for each sample (Figures S2–S6) and the distribution of TPM values in each sample (Table S4). Additionally, a snapshot from the IGV Genome Browser was included (Figure S7). Quality control measures included the expression value distribution across the sample set (Figure S8), groupwise correlation values for spike-ins (Table S5) and genomic reads (Table S6), Sample correlations for spike-in reads (Figure S9), sample correlations for genomic reads (Figure S10), sample correlations for genomic reads without tissue samples (Figure S11), hierarchical clustering for spike-in reads (Figure S12) and genomic reads (Figure S13), as well as PCA plots for spike-in reads (Figure S14) and genomic reads (Figure S15).

### 3.2. Differential Expression Analyses

**Figure 4:**
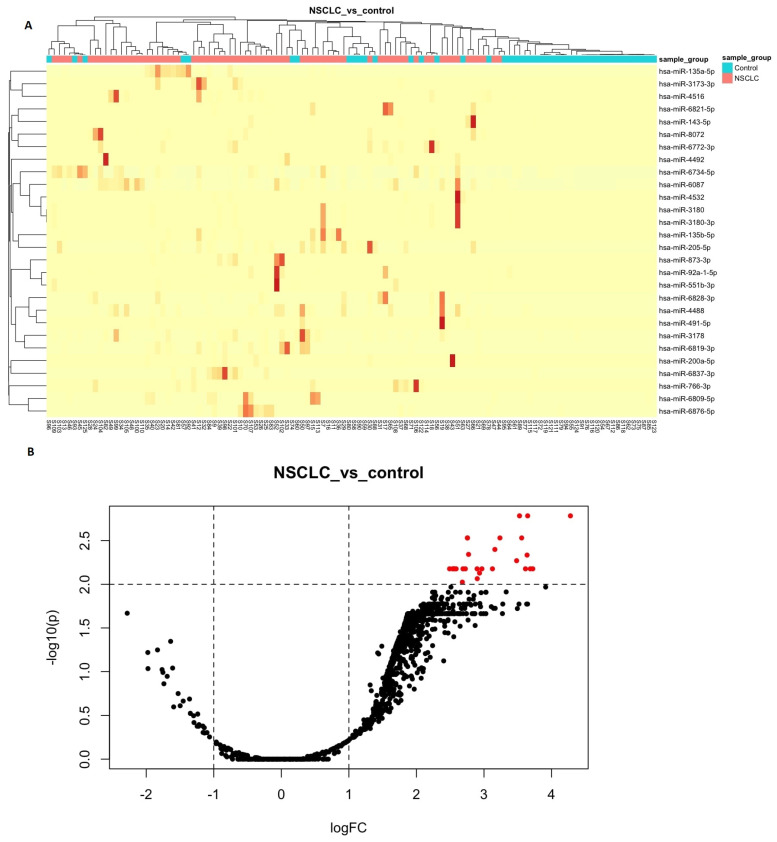
(**A**) Heatmap comparing NSCLC with the control group. Hierarchical clustering of samples was performed using the Pearson correlation coefficient, and features were filtered based on the similarity of their expression patterns. In the heatmap, high expression is indicated by red color while low expression is indicated by yellow color. Each row represents a differentially expressed feature, and each column represents a sample. The heatmap exhibits a wide range of values in the TMP (normalized values) cells, ranging from 0 to, on some occasions, a few thousand. Scaling the data further poses a challenge, given the existing row scaling. The data table used to generate the heatmap is included as a supplementary resource (Table S8). (**B**) Volcano plot comparing NSCLC vs. control. The *y*–axis represents the log10 of the *p*–values, while the *x*–axis represents the logFC calculated for the comparison group vs. the baseline group. The plot illustrates the behavior of the reliability values of the measurement characteristics in relation to the fold change. The filtering thresholds used are marked with dashed lines in the plot, and upregulated genes are colored red while downregulated genes are not detected.

Using loose filtering criteria, we identified 690 upregulated miRNAs and 2 downregulated miRNAs (hsa-miR-32-5p and hsa-miR-3613-5p) in NSCLC compared to the control group. After refining the filtering criteria, we found 28 upregulated miRNAs and no downregulated ones. Detailed information regarding the characteristics of these 28 upregulated miRNAs can be found in Table 2 and Figure 4A,B. Additionally, Table S7 provides additional statistically significant parameters.

Furthermore, comprehensive comparative analyses were conducted among subgroups of both cancer and non-cancer patients. A comprehensive summary of the results obtained from these individual comparisons, considering the differentially expressed (DE) miRNAs, can be found in Table S9. 

Multiple pairwise comparisons revealed the presence of shared differentially expressed (DE) miRNAs among different subgroups. Specifically, nine miRNAs (hsa-let-7a-2-3p, hsa-miR-103a-2-5p, hsa-miR-105-5p, hsa-miR-1178-3p, hsa-miR-1180-5p, hsa-miR-1208, hsa-miR-1225-3p, hsa-miR-1225-5p, hsa-miR-1227-3p) were found to be upregulated in three comparisons: AC vs. COPD, AC vs. noCOPD, and AC vs. SCC. In addition, hsa-miR-202-3p was found to be upregulated in both NSCLC vs. noCOPD and COPD vs. noCOPD, and three miRNAs (hsa-miR-3173-3p, hsa-miR-6819-3p, and hsa-miR-6821-5p) were upregulated in both NSCLC vs. COPD and NSCLC vs. noCOPD. These shared DE miRNAs are listed in Table S10. 

### 3.3. Enrichment Analysis for the Differentially Expressed miRNA

Functional enrichment analysis revealed that the differentially expressed (DE) miRNAs in the NSCLC group were significantly enriched in various biological processes, molecular functions, cellular components, and pathways involved in biological interactions. Several overrepresented GO terms potentially implicated in the pathogenesis of NSCLC were identified, including the cellular nitrogen compound metabolic process, gene expression, and biosynthetic process. In addition, KEGG pathway analysis identified potentially carcinogenesis-related pathways, such as fatty acid biosynthesis, adherens junctions, and the p53 pathway. The enrichment test results are ranked by *p*–value for each GO term and KEGG pathway and are presented in Table 3, which shows GO biological processes, molecular functions, and cellular components, as well as KEGG pathways for biological interactions. Additional comprehensive details and supplementary analyses can be accessed in Tables S11–S14. 

### 3.4. Gradient Boosting Decision Tree to Determine Diagnostic Value of Serum miRNAs in NSCLC Patients

**Figure 5:**
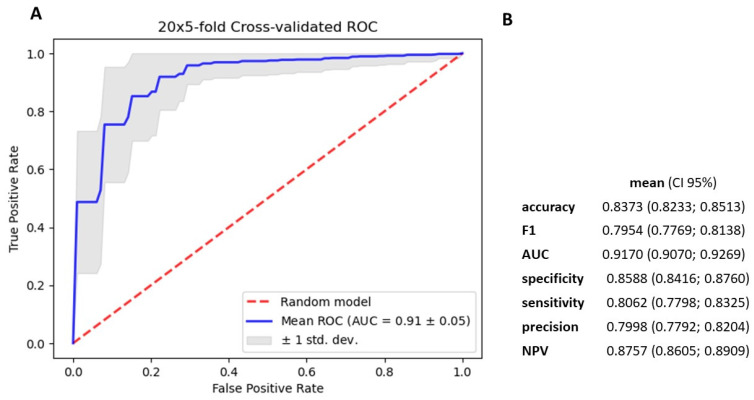
Metrics for gradient boosting decision tree model based on 2588 miRNA. (**A**). mean ROC ± SD curve, and mean AUC for classifier (**B**). mean and 95% CI of accuracy, f1–score metrics, AUC, specificity, sensitivity, precision and NPV for differentiating cancerous and noncancerous patients serum.

**Figure 6:**
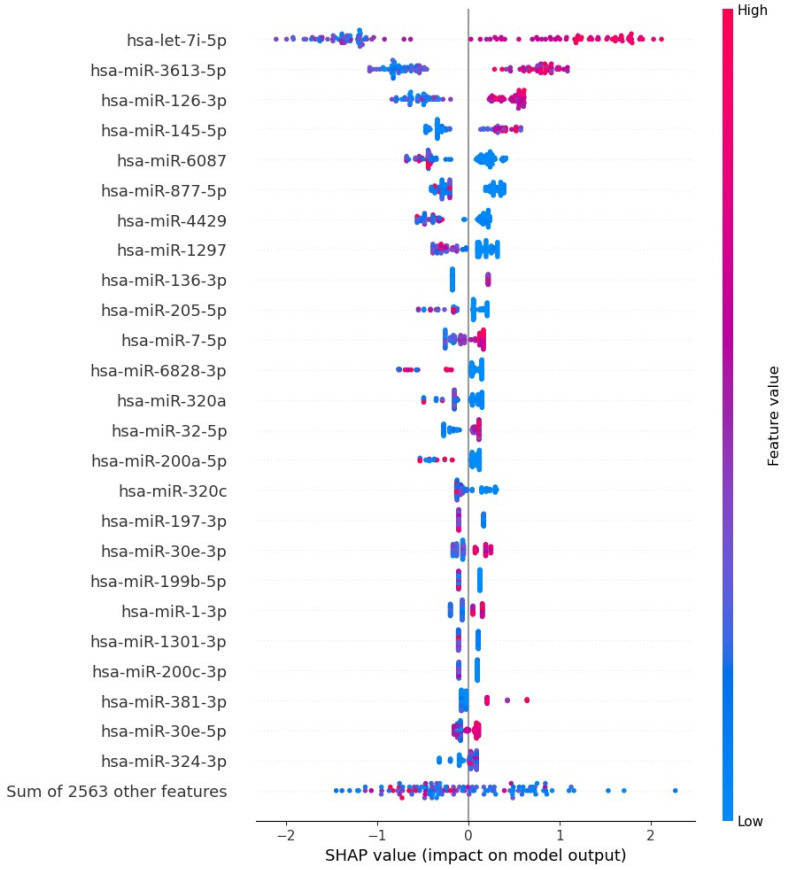
Shapley Additive Explanation for the Gradient Boosting Decision Tree Classifier. The *y*–axis indicates a ranking of variables, values of miRNA, sorted from the most important in the model (top) to the least important (bottom). In the figure, we named the 25 most important miRNA in the model. The *x*–axis indicates an impact of a given variable on the model’s predictions; the SHAP values are sorted from the negative impact leading towards the cancer (class 0 on the left) to the positive impact leading towards the non-cancer (class 1 on the right). There are 118 points per row, one point per patient, where each indicates an attribution of a given variable to the probability model output. The color–axis indicates the variables’ values from low with blue to high with red. The visible distinction in colors between negative and positive SHAP values might be viewed as indicating a significant expression profile (up or down).

**Figure 7:**
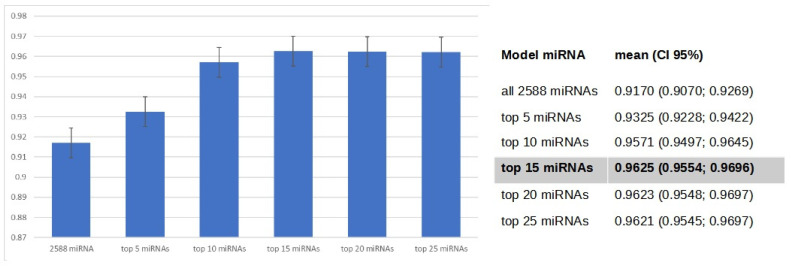
AUC mean and 95%CI of gradient boosting decision tree models with selected miRNAs.

**Figure 8:**
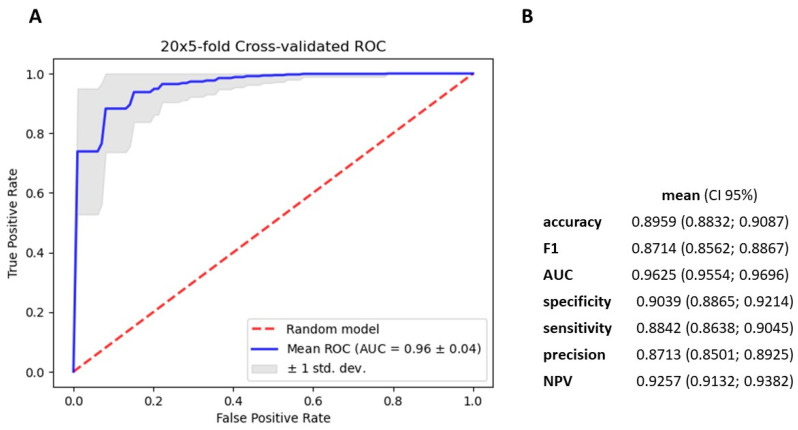
Metrics for gradient boosting decision tree classifier based on the 15 miRNA that were the most important in the model. (**A**) mean ROC ± SD curve, and mean AUC for classifier (**B**) mean and 95% CI of accuracy, f1–score metrics, AUC, specificity, sensitivity, precision and NPV for differentiating cancerous and noncancerous patients serum.

We utilized the TMM normalization method to obtain normalized gene counts, which served as the basis for constructing a gradient boosting decision tree classifier (Table S15). This classifier represents an advanced machine learning algorithm that holds immense promise in the field of medical science. Our predictive model demonstrated high diagnostic potential in distinguishing NSCLC from non-cancerous patients, achieving an AUC value of 0.91 ± 0.05, sensitivity of 0.806, and specificity of 0.859, based on the analysis of 2588 serum miRNA values (Figure 5). Using Shapley Additive exPlanations (SHAP), we identified the 25 miRNAs with the strongest impact on the model (Figure 6). We further developed five simplified models based on SHAP, using the top 5, 10, 15, 20, and 25 miRNAs. Among these models, the best performance was observed with the top 15 impacted miRNAs (AUC = 0.9625 ± 0.04; Figure 7 and Figure 8). This group consists of eight downregulated miRNAs (hsa-let-7i-5p, hsa-miR-3613-5p, hsa-miR-126-3p, hsa-miR-145-5p, hsa-miR-136-3p, hsa-miR-7-5p, hsa-miR-320a, hsa-miR-32-5p) and seven upregulated miRNAs (hsa-miR-6087, hsa-miR-877-5p, hsa-miR-4429, hsa-miR-1297, hsa-miR-205-5p, hsa-miR-6828-3p, hsa-miR-200a-5p). These results suggest that the selected panel of 15 miRNAs holds promise as a novel and valuable diagnostic tool for distinguishing between lung malignant and non-malignant patients (Figure 8).

## 4. Discussion

In this study, we conducted a global miRNA profiling analysis on liquid biopsy samples from early-stage NSCLC patients and non-cancerous pulmonary controls using NGS. Our objective was to identify a serum miRNA signature with high accuracy for discriminating NSCLC from non-cancer patients and to investigate the functional implications of the serum miRNA expression profile for understanding the biological pathways and molecular mechanisms involved in NSCLC development and progression.

Serum miRNAs have several advantages over other biomarkers, such as tissue biopsies or circulating tumor cells (CTCs), for cancer detection and monitoring. They are stable, abundant, and easily accessible in blood samples, and can reflect the heterogeneity and dynamics of tumor cells and their microenvironment [28,29]. Previous studies have reported various serum miRNA signatures for NSCLC diagnosis, prognosis, and response to therapy. However, most of these studies used small sample sizes, different platforms, or different normalization methods, which may limit the reproducibility and comparability of the results [13,30,31,32]. 

To address these limitations, we utilized a well-characterized cohort of 71 early-stage NSCLC patients and 47 non-cancerous controls and performed global miRNA profiling using NGS, a highly sensitive and accurate method for miRNA detection and quantification [24]. Additionally, we implemented stringent quality control steps during serum processing to assess hemolysis, RNA purification, and cDNA synthesis in order to identify and exclude any outliers. Furthermore, we applied rigorous statistical analysis and preprocessing steps to ensure the reliability and robustness of our findings. 

We constructed a gradient-boosting decision tree classifier that accurately distinguishes NSCLC from non-cancerous patients. The analysis of 2588 serum miRNA values revealed their significant discriminatory capabilities achieving an AUC value of 0.91 ± 0.05 (sensitivity 0.806, specificity 0.859). Employing SHAP, we identified the top 25 miRNAs, and the 15 exhibited the strongest discriminatory potential were used to create a simplified GBDT model, resulting in an AUC value of 0.96 ± 0.04 (sensitivity 0.884, specificity 0.903). Among the selected 15 miRNAs, 8 were downregulated and 7 were upregulated in NSCLC. These findings underscore the promise of our approach in uncovering miRNA biomarkers for diagnosing NSCLC and open avenues for further exploring the functional relevance of these identified miRNAs in the context of lung cancer. The diagnostic performance of our model was comparable or even better than those presented in the literature of the last years.

Numerous studies have assessed the diagnostic potential of individual miRNAs included in our signature, specifically miR-126-3p, miR-145-5p, miR-7-5p, miR-6087, miR-4429, and miR-205-5p. Moving on to Wang et al.’s findings [28], they reported that serum miR-126-3p exhibits early detection capabilities for NSCLC patients, with sensitivity and specificity comparable to traditional tumor markers. Moreover, Soliman et al. [33] found that miR-126-3p is significantly downregulated in the serum of NSCLC patients compared to healthy controls, offering high sensitivity and specificity (AUC: 0.90) in distinguishing NSCLC patients from controls. Furthermore, Gan et al. [34] demonstrated that miR-145-5p is markedly downregulated in NSCLC tissues and serum compared to healthy tissues and serum, with high sensitivity and specificity (AUC: 0.88) in distinguishing NSCLC patients from healthy controls. Additionally, low miR-145-5p expression correlated with poor overall survival and disease-free survival in NSCLC patients. Shifting focus to Petkova et al.’s research [35], they showed that miR-7-5p is upregulated in both lung adenocarcinoma and squamous cell lung cancer compared to normal tissues, suggesting its potential as a diagnostic biomarker for NSCLC due to its association with clinical outcomes and tumor subtypes. Similarly, Kumar et al. [36] reported significantly lower miR-320a expression in the serum of NSCLC patients compared to controls, with miR-320a exhibiting high diagnostic performance for NSCLC, featuring an AUC of 0.844. In addition, Liu et al. [37] demonstrated significantly higher miR-6087 expression in the serum of NSCLC patients compared to controls, with a high diagnostic performance for NSCLC (AUC: 0.780) when combined with miR-4687-3p using a logistic regression model. Turning to Ruan et al.’s investigation [38], they showed that miR-4429 expression was significantly lower in the serum of NSCLC patients compared to controls and correlated with tumor size, EGFR mutation, lymph node metastasis, and TNM stage. Remarkably, miR-4429 exhibited high diagnostic performance for NSCLC, featuring an AUC of 0.918, with sensitivity and specificity of 89.34% and 84.72%, respectively. Additionally, Wang et al. [39] reported that miR-4429 expression was significantly lower in the serum of NSCLC patients compared to controls, with a high diagnostic performance (AUC: 0.898), sensitivity of 87.5%, and specificity of 82.81%. Furthermore, Zhao et al. [40] highlighted miR-205-5p’s overexpression in NSCLC tissues and serum, suggesting its potential as a biomarker for NSCLC diagnosis (AUC: 0.8250). Lastly, Jiang et al. [41] demonstrated that miR-205-5p is highly expressed in NSCLC tissues and serum, particularly in the squamous cell carcinoma subtype, and can effectively differentiate NSCLC from benign lung diseases and healthy controls.

Serious of studies have underscored the diagnostic potential of specific miRNAs, including miR-126-3p, miR-145-5p, miR-7-5p, miR-6087, miR-4429, and miR-205-5p, within our signature. These miRNAs exhibit promise as valuable biomarkers for the early detection and differentiation of NSCLC from healthy controls. Their high sensitivity and specificity, as demonstrated in various research studies, highlight their potential to enhance the accuracy of NSCLC diagnosis and contribute to improved patient outcomes. Further research and validation are warranted to solidify their clinical utility in NSCLC diagnostics. 

Prior to our studies, other researchers had been working to develop miRNA-based diagnostic signatures for NSCLC. Using qPCR, Yang et al. [32] tested the potential of a panel of four circulating miRNAs (hsa-miR-146b, hsa-miR-205, hsa-miR-29c, and hsa-miR-30b) as biomarkers for early diagnosis of NSCLC. They found that these miRNAs were significantly elevated in serum samples of NSCLC patients compared to healthy or cancer-free controls, and that they had high accuracy (AUC = 0.96) and sensitivity (0.92) for distinguishing NSCLC from controls. The panel also showed better performance for detecting adenocarcinoma than squamous cell carcinoma and reflected the tumor stage and load. Moreover, two of the miRNAs (hsa-miR-146b and hsa-miR-29c) were associated with poor survival outcomes, especially for squamous cell carcinoma patients. The authors concluded that the 4-miRNA panel is a novel, sensitive and non-invasive serum marker for the early diagnosis of NSCLC.

Additional studies have also explored the diagnostic potential of miRNAs signature in NSCLC. Using qPCR, Ying et al. [42] identified a panel of five miRNAs with high sensitivity (83.0%) and specificity (90.7%) for detecting NSCLC regardless of smoking status, gender, and ethnicity. Zhu et al. [43] examined miRNA by using qPCR and developed a classifier based on four miRNAs that could distinguish lung cancer from other conditions with high AUC (0.885). Using NGS, Duan et al. [44] identified a set of three miRNAs that were significantly increased in NSCLC patients and had an AUC of 0.828, sensitivity of 86.7%, and specificity of 71.7%. Wang et al. [45] using the TaqMan Low Density Array and qPCR identified a panel of five serum miRNAs that exhibited high AUC values (0.976 and 0.823) for detecting NSCLC, particularly in early stages. Masayasu et al. [46] used automated machine learning on NGS results to construct and screen 1123 miRNA-based diagnostic models for lung cancer detection. The best model showed an AUC of 0.98, sensitivity of 85.7%, and specificity of 92.9%. They also compared their model with CEA, a conventional blood biomarker for adenocarcinoma, and found that their model had higher sensitivity for early-stage lung adenocarcinoma.

When evaluating the diagnostic efficacy of our miRNA panel in comparison to the presently employed clinical biomarkers for NSCLC, our panel exhibits remarkable performance metrics, showcasing a high level of accuracy (0.896), sensitivity (0.884), and specificity (0.903). These results not only meet but frequently surpass the performance of established diagnostic biomarkers commonly utilized in this field, including carcinoembryonic antigen (CEA) [47], cytokeratin 19 fragment (CYFRA 21-1) [48], neuron-specific enolase (NSE), squamous cell carcinoma antigen (SCC) [49], tissue polypeptide antigen (TPA) [50], and matrix metalloproteinase (MMP-9) [51]. In contrast to our miRNA panel, conventional biomarkers such as CEA, NSE, and MMP-9 exhibit multiple limitations. They suffer from suboptimal sensitivity and specificity, typically ranging from 50% to 80% [52,53,54]. Furthermore, they lack standardization and validation, resulting in variability across different laboratories and methodologies. These biomarkers are also susceptible to confounding factors, including smoking, inflammation, tumor stage, histology, smoking status, and comorbidities. Additionally, they lack specificity for NSCLC, often showing elevated levels in other cancer types or benign conditions. For example, a study conducted by Xu et al. [54] reported diagnostic sensitivity, specificity, and AUC of 80.0%, 72.2%, and 0.84, respectively, for serum CEA, 71.0%, 83.3%, and 0.80 for NSE, respectively, and 87.1%, 80.56%, and 0.89 for MMP-9, respectively. While these biomarkers may demonstrate elevated levels in NSCLC patients, their diagnostic performance falls short of the capabilities of our miRNA panel. Moreover, Ajona et al. [55] introduced a diagnostic model based on the quantification of complement-derived fragment C4c, CYFRA 21–1, and C-reactive protein (CRP) in plasma. Their model exhibited commendable specificity (92%) in distinguishing between benign and malignant pulmonary nodules with an AUC of 0.86. Our miRNA panel exhibited a specificity level akin to theirs, registering at 0.903.

As demonstrated, our miRNA panel equals or surpasses the currently employed clinical biomarkers for NSCLC diagnosis, offering a promising opportunity to establish a reliable and efficient diagnostic tool for detecting NSCLC that effectively addresses the limitations associated with traditional biomarkers and holds the possibility of enhancing patient outcomes.

The potential value of miRNAs as biomarkers for the early detection of NSCLC is underscored by existing literature and our own investigations. The diagnostic test based on miRNAs offers a promising complement to histopathological evaluation with the potential to enhance the accuracy and efficiency of NSCLC screening. This improvement in screening efficacy holds the promise of enabling earlier interventions and ultimately improving patient outcomes. However, to fully integrate the miRNA-based diagnostic model into routine clinical practice, additional research and validation are necessary. 

Furthermore, we identified a set of 28 significantly upregulated DEmiRNAs in NSCLC samples when compared to controls. Among the top 10 upregulated miRNAs, some have been previously reported to be associated with NSCLC or other cancers, while others have not been extensively studied. Our findings are consistent with some previous reports, such as the upregulation of hsa-miR-4488, hsa-miR-205-5p, hsa-miR-92a-1-5p, and hsa-miR-551b-3p [37,40,56,57,58,59,60,61,62]. However, our results differ from previous studies regarding hsa-miR-3180-3p and hsa-miR-3178, which were found to be downregulated [63,64,65,66,67]. Furthermore, expression of hsa-miR-6819-3p, hsa-miR-6734-5p, hsa-miR-4492, and hsa-miR-3180 in NSCLC has not been thoroughly investigated.

Liu et al. [37] previously identified hsa-miR-4488, which was the most upregulated miRNA in our study, as one of the six differentially expressed miRNAs in serum NSCLC. Additionally, hsa-miR-4488 was found to be a potential biomarker for breast cancer progression and metastasis, as well as a suppressor of angiogenesis by directly targeting CX3CL1 in a study by Zheng et al. [56]. Zhao et al. [40] demonstrated that hsa-miR-205-5p, which was the second most upregulated miRNA in our study, was overexpressed in NSCLC tissues and cell lines and promoted lung cancer cell growth and invasion by downregulating TP53INP1, consequently modulating the levels of P21, RB1, and cyclin D1. Furthermore, Zhu et al. [57] showed that hsa-miR-205-5p increased cancer cell proliferation, migration, invasion, and cell cycle progression by activating the PTEN/PI3K/AKT signaling pathway. Hsa-miR-92a, which was the fourth most upregulated miRNA in our study, was found to be overexpressed in NSCLC tissues and cell lines, and implicated in promoting epithelial–mesenchymal transition (EMT) by activating the PTEN/PI3K/AKT signaling pathway, according to a study by Liu et al. [58]. Additionally, Yu et al. [59] reported that hsa-miR-92a-1-5p was overexpressed in extracellular vesicles of prostate cancer patients and promoted osteoclast differentiation by reducing MAPK1 and FoxO1 expression. Hsa-miR-551b-3p, which was the eighth most upregulated miRNA in our study, was found to be overexpressed in extracellular vesicles (EVs) released by multidrug-resistant (MDR) NSCLC cells, as reported by Sousa et al. [60]. Furthermore, Karanam et al. [61] showed that hsa-miR-551b-3p promoted tumor growth, invasion, and metastasis by targeting GLIPR2 in high-risk head and neck cancer, and Chang et al. [62] reported that hsa-miR-551b-3p targeted cyclin D1 and inhibited tumor growth in cholangiocarcinoma. Based on the aforementioned evidence, we hypothesize that hsa-miR-4488, hsa-miR-205-5p, hsa-miR-92a-1-5p, and hsa-miR-551b-3p may act as oncogenic miRNAs in NSCLC by targeting tumor suppressor genes or pathways, although their role may vary among different cancers.

In a previous study, Chen et al. [63] found that hsa-miR-3180-3p, the fifth most upregulated miRNA in our study, was downregulated in exosomes derived from A549 cells. Moreover, they demonstrated that hsa-miR-3180-3p inhibited proliferation and metastasis of NSCLC by downregulating FOXP4, a transcription factor that promotes tumor growth and invasion [63]. In another study, Jin et al. [64] found that serum hsa-miR-3180-3p was downregulated in gastric cancer patients with cisplatin resistance, suggesting that it may function as a prognostic biomarker. Similarly, He et al. [65] found that hsa-miR-3178, the ninth most upregulated miRNA in our study, was downregulated in the plasma of NSCLC patients compared to healthy controls. Interestingly, Wang et al. [66] reported that overexpression of hsa-miR-3178 inhibited migration and invasion of highly metastatic prostate, lung, and breast cancer cells under in vitro conditions, whereas antagonizing hsa-miR-3178 promoted those events in their lowly metastatic counterparts. Furthermore, their findings revealed a significant decrease in hsa-miR-3178 expression in cancer cell lines with high metastatic capacity compared to their lowly metastatic counterparts. Wu et al. [67] found that hsa-miR-3178 was downregulated in gastric cancer tissues and cells, which was significantly associated with the TNM stage and lymph node metastasis of patients and a poor prognosis. Therefore, we hypothesize that hsa-miR-3180-3p and hsa-miR-3178 may exhibit distinct roles in the same cancer depending on the stage and the biological material or have diverse roles and targets in various types of cancer cells. Although the biological function and expression levels of hsa-miR-3180-3p and hsa-miR-3178 in NSCLC have not been extensively investigated and fully elucidated, the available findings and existing knowledge regarding the behavior of miRNAs in cancer provide a basis for our hypothesis. We suggest that hsa-miR-3180-3p and hsa-miR-3178 may display distinct roles within the same cancer type, potentially functioning as either tumor suppressors or oncogenes. These roles could vary depending on the specific stage of the cancer, metastatic potential of the cells, and the nature of the biological material under investigation, such as liquid biopsy, tissue samples, or cell cultures [68]. However, further research is imperative to validate these hypotheses and to establish a comprehensive understanding of the precise functions and underlying mechanisms of these miRNAs in the context of NSCLC.

The mechanisms and functions of hsa-miR-6819-3p, hsa-miR-6734-5p, hsa-miR-4492, and hsa-miR-3180 in NSCLC remain unknown. Gao et al. [69] predicted that hsa-miR-6819-3p, the third most upregulated miRNA in our study, may promote tumor growth by targeting ACTG1 and being upregulated in alcohol-associated hepatocellular carcinoma (HCC) tissues compared to non-alcohol-associated HCC tissues. Muwonge et al. [70] found that hsa-miR-6819-3p was significantly upregulated in the serum of patients with epidemic Kaposi’s sarcoma (KS) compared to HIV-positive patients without KS, indicating its potential as a biomarker for KS. Wan et al. [71] reported that hsa-miR-6734-5p, the sixth most upregulated miRNA in our study, is associated with high-grade serous ovarian cancer. Additionally, hsa-miR-4492, the seventh most upregulated miRNA in our study, is frequently silenced by lncRNAs in ovarian and bladder cancer, specifically FOXD2-AS1 and LINC00319, respectively, which thus promotes proliferation, migration, and invasion [72,73]. Meanwhile, hsa-miR-3180, the tenth most upregulated miRNA in our study, is modulated by lncKRT16P6 in tongue squamous cell carcinoma [74] and by SNHG17 in hepatocellular carcinoma [75]. Furthermore, hsa-miR-3180 has been shown to be a critical regulator involved in de novo fatty acid synthesis and uptake in HCC [76].

There are several reasons why our findings for miRNA expression (e.g., hsa-miR-3180-3p and hsa-miR-3178) may not be consistent with those reported in previous studies. First, different sources and types of samples, such as tissues, blood, plasma, serum, exosomes, and other body fluids, may yield varying miRNA profiles. Each of these sample types may yield different miRNA profiles due to variations in miRNA release mechanisms [77]. Furthermore, it is crucial to acknowledge that miRNAs, which target proto-oncogenes and tumor suppressor genes, undergo regulation within cancer cells based on the specific context imposed by the ongoing process of carcinogenesis. This implies that the expression patterns of miRNAs in tumor tissue may carry a distinct connotation compared to the profiles observed in the cell-free circulating miRNAs we analyzed from blood serum [78,79]. Additionally, different subtypes of NSCLC, such as adenocarcinoma or squamous cell carcinoma, may also exhibit distinct miRNA expression patterns [78,79]. Second, different detection methods and platforms, including microarray, qRT-PCR, and sequencing, can affect the sensitivity, specificity, and reproducibility of miRNA measurement [78,79]. Third, patient characteristics and clinical factors, such as age, gender, smoking status, ethnicity, stage, grade, histology, treatment, and prognosis, may impact the biogenesis, stability, and function of miRNAs [78,79,80]. Finally, environmental and lifestyle factors, such as carcinogens, pollutants, diet, exercise, stress, and infection, may also alter miRNA expression through epigenetic mechanisms, directly or indirectly [80]. Therefore, consideration and validation of these factors are essential for interpreting and comparing miRNA expression data across studies.

To identify the biological processes and pathways associated with NSCLC development and progression regulated by the serum miRNAs, we conducted a pathway enrichment analysis based on the target genes of the 28 serum miRNAs. Our analysis revealed that some of the serum miRNAs were involved in key metabolic, structural, and signaling pathways in NSCLC, including fatty acid biosynthesis (regulated by 3 miRNAs), adherens junctions (regulated by 12 miRNAs), and the p53 pathway (regulated by 13 miRNAs). These pathways play critical roles in the regulation of fundamental cellular events in NSCLC pathogenesis, such as cell cycle, apoptosis, differentiation, stemness, EMT, and inflammation [81,82,83,84]. For example, fatty acid biosynthesis is crucial for providing energy and membrane components for cancer cell growth and survival. Fatty acid synthase (FASN), the key enzyme in this pathway, is overexpressed in NSCLC, and inhibiting its expression can suppress tumor growth and induce apoptosis [81]. Adherens junctions are essential for maintaining cell–cell adhesion and polarity, and their disruption can facilitate EMT and metastasis. In NSCLC, E-cadherin, a major component of adherens junctions, is downregulated, and its expression correlates with tumor differentiation and prognosis [82]. The p53 pathway is a significant tumor suppressor pathway that mediates DNA damage response and apoptosis in cancer cells [83]. Mutations or inactivation of the p53 pathway are common in NSCLC, leading to resistance to chemotherapy and radiotherapy [84]. However, it is important to note that our findings are currently based solely on statistical associations. To confirm and strengthen our results, validation is required.

Our study has illuminated the potential value of a non-invasive serum miRNA signature as a supplementary tool for early NSCLC detection. However, it is vital to recognize the limitations that call for attention in future research endeavors. Firstly, our study’s sample size was relatively modest, potentially lacking the ability to fully capture the diverse NSCLC patient population. Secondly, our control group consisted of individuals affected by non-cancerous pulmonary conditions, including chronic obstructive pulmonary disease, pneumonia, and bronchitis. It is noteworthy that such conditions can influence serum miRNA expression. Therefore, a more appropriate approach would involve comparing our miRNA panel against healthy individuals. Thirdly, our miRNA panel was developed using data solely from a single next-generation sequencing platform. This singular platform approach may introduce technical biases and unwarranted variability. To mitigate these concerns, it is imperative to validate our miRNA panel using diverse platforms or methodologies, such as quantitative real-time PCR or microarray analysis. Lastly, the development of our miRNA panel relied on a supervised machine learning algorithm, which carries the risk of overfitting data and potentially compromising the model’s generalizability. Consequently, it is advisable to incorporate cross-validation techniques or utilize independent test sets to thoroughly assess the robustness and stability of our miRNA panel in future investigations. To address these concerns, we are actively engaged in the recruitment of additional patients and have plans to include a larger and more representative control group. Furthermore, we intend to perform validation using quantitative real-time PCR (qPCR). These steps are vital to ensure the reliability and generalizability of our findings, thereby strengthening the overall robustness of our study.

Circulating miRNAs have garnered substantial attention from the scientific and clinical communities due to their relevance in various ongoing clinical investigations, particularly within the context of cancer, including non-small cell lung cancer (NSCLC). A notable study, registered under the identifier NCT04427475, is focused on elucidating the diagnostic potential of plasma exosomal miRNAs, both prior to and following immunotherapy targeting PD-1 or PD-L1 in NSCLC patients. The primary objectives of this investigation are to unveil alterations in the PD-L1 and miRNA expression profiles within exosomes in response to immunotherapeutic interventions, as well as to assess the viability of plasma exosomal PD-L1 and miRNAs as prospective biomarkers for predicting the therapeutic efficacy of anti-PD-1/PD-L1 treatment in NSCLC patients [85]. Furthermore, an additional study, registered as NCT02247453, aims to identify novel diagnostic miRNAs within plasma samples sourced from individuals afflicted with lung cancer, utilizing advanced next-generation sequencing techniques. The overarching goal of this study is to demonstrate the efficacy of plasma miRNA profiling as a primary screening method for the early detection of lung cancer. By doing so, it aspires to reduce the unnecessary utilization of low-dose computed tomography scans, thus optimizing the diagnostic process for this malignancy [85].

The utilization of liquid biopsy-based miRNA profiling presents a compelling approach that offers a non-invasive and real-time method for detecting and characterizing early-stage NSCLC. This approach has the potential to effectively complement routine histopathology, especially in cases with diagnostic ambiguity. Furthermore, it holds the promise of not only complementing but also potentially replacing existing imaging-based methods, which are often costly and time-consuming. Given the prevalent late-stage diagnosis of NSCLC, which severely limits treatment options and compromises overall survival rates, early detection becomes crucial. Furthermore, our findings could have the potential to serve as a step towards conducting comprehensive investigations into the specific roles of selected miRNAs in NSCLC carcinogenesis under tightly controlled in vitro conditions. This foundational research can pave the way for subsequent efforts aimed at precisely identifying promising therapeutic targets, which in turn can contribute to the development of more effective treatments. Nonetheless, to ensure the clinical applicability and significance of our findings, it is crucial to conduct validation studies involving independent cohorts of patients; a course of action that is already planned for the near future.

## 5. Conclusions

In this study, we utilized NGS technology to perform global miRNA profiling analysis on liquid biopsy samples obtained from early-stage NSCLC patients and controls (non-cancerous pulmonary patients). The primary objectives were to identify a diagnostic serum miRNA signature for NSCLC and to establish differentially expressed miRNAs (DEmiRNAs). Additionally, we explored the functional implications of the serum miRNA expression profile in the development and progression of NSCLC. To ensure the reliability and robustness of our findings, our study employed a well-characterized cohort and implemented stringent quality control measures.

We demonstrated the effectiveness of the gradient-boosting decision tree classifier, an advanced machine-learning algorithm, in accurately distinguishing NSCLC from non-cancerous patients using the top 15 miRNAs with the strongest discriminatory potential, yielding significant AUC values of 0.96 ± 0.04. Additionally, our study showed 28 significantly upregulated miRNAs in NSCLC samples compared to controls. Notably, some of these miRNAs have been previously associated with NSCLC and other cancers, while others remain relatively unexplored. To gain deeper insights, we performed pathway enrichment analysis, revealing the involvement of miRNAs in key metabolic, structural, and signaling pathways in NSCLC, such as fatty acid biosynthesis, adherens junctions, and the p53 pathway. These pathways play critical roles in NSCLC pathogenesis and provide potential targets for therapeutic interventions. While the findings were consistent with some previous reports, they also differed from others, emphasizing the need for further investigation and validation.

The identification of serum miRNAs signature for detection of early-stage NSCLC patients holds significant implications for the diagnosis and potential treatment of the disease. Liquid biopsy-based miRNA profiling offers a non-invasive and real-time approach for early detection that complements existing histopathology and imaging methods. This integrated approach has the potential to enhance diagnostic accuracy, facilitate timely interventions, and ultimately improve patient outcomes by enabling personalized treatment strategies.

## Figures and Tables

**Table 1:** Patient’s characteristics.

Patient’s Characteristics		
Study group	*n*	71
Age (years)	Mean ± SD *	65.59 ± 6.91
	Median	65
	Range	49–81
Sex	Female	31 (43.7%)
	Male	40 (56.3%)
Tumor stage	IA	20 (28.2%)
	IB	19 (26.8%)
	IIA	13 (18.3%)
	IIB	9 (12.7%)
	IIIA	10 (14.1%)
Histology	SCC	36 (50.7%)
	AC	32 (45.1%)
	LCC	2 (2.8%)
	NSCLC NOS	1 (1.4%)
Smoking		66 (93%)
Control group	*n*	47
Age (years)	Mean ± SD *	64.19 ± 9.67
	Median	65
	Range	37–83
Sex	Female	17 (36.2%)
	Male	30 (63.8%)
Diagnosis	COPD	21 (44.7%)
	Emphysema	1 (2.1%)
	Bronchitis	2 (4.3%)
	Pneumonia	1 (2.1%)
	Fibroma	1 (2.1%)
	Metabolically active proliferative process	1 (2.1%)
	Sarcoidosis	1 (2.1%)
	Chronic cough	1 (2.1%)
	Lower respiratory symptoms	18 (38.3%)
Smoking		47 (100%)
All patients	*n*	118
Age (years)	Mean ± SD *	65.03 ± 8.11
	Median	65
	Range	37–83
Sex	Female	48 (40.7%)
	Male	70 (59.3%)
Smoking		113 (95.8%)

Legend: * SD (Standard Deviation).

**Table 2:** List of 28 miRNAs that were upregulated in NSCLC vs. control.

ID	logFC	FDR adj.P.Val	avgRank
hsa-miR-4488	4.28	0.00165	1
hsa-miR-205-5p	3.65	0.00165	2
hsa-miR-6819-3p	3.53	0.00165	3
hsa-miR-92a-1-5p	3.56	0.00295	4
hsa-miR-3180-3p	3.64	0.00463	5
hsa-miR-6734-5p	3.72	0.00665	6
hsa-miR-4492	3.69	0.00665	7
hsa-miR-551b-3p	3.61	0.00665	8
hsa-miR-3178	3.24	0.00295	9
hsa-miR-3180	3.48	0.00537	10
hsa-miR-6821-5p	3.16	0.00399	11
hsa-miR-8072	3.13	0.00665	13
hsa-miR-491-5p	2.97	0.00665	15
hsa-miR-873-3p	2.90	0.00665	16
hsa-miR-200a-5p	2.75	0.00295	17
hsa-miR-3173-3p	2.77	0.00455	18
hsa-miR-6087	2.76	0.00295	19
hsa-miR-4516	2.93	0.00744	20
hsa-miR-766-3p	2.90	0.00862	22
hsa-miR-4532	2.72	0.00665	27
hsa-miR-135a-5p	2.69	0.00665	31
hsa-miR-6772-3p	2.68	0.00945	35
hsa-miR-143-5p	2.59	0.00665	36
hsa-miR-6876-5p	2.57	0.00665	39
hsa-miR-6837-3p	2.55	0.00665	40
hsa-miR-6828-3p	2.54	0.00665	41
hsa-miR-135b-5p	2.49	0.00665	43
hsa-miR-6809-5p	2.55	0.00665	44

Legend: Average ranking value based on both *p* value and fold change; value 1 is the strongest DE feature.

**Table 3:** Gene Ontology (GO) and KEGG pathway enrichment analysis results for differentially expressed miRNAs in NSCLC vs. control.

GO Category—Biological Processes	*p*-Value	No. of Genes	No. of miRNAs
Cellular nitrogen compound metabolic process	4.23 × 10^−132^	971	14
Gene expression	4.60 × 10^−101^	229	13
Biosynthetic process	5.66 × 10^−93^	806	15
Viral process	1.60 × 10^−58^	159	14
Symbiosis, encompassing mutualism through parasitism	5.42 × 10^−58^	171	14
Cellular protein modification process	6.23 × 10^−51^	463	15
Biological process	2.92 × 10^−44^	2423	16
Catabolic process	6.18 × 10^−43^	393	14
Small molecule metabolic process	2.38 × 10^−39^	433	14
**GO Category—Molecular Functions**	***p*-Value**	**No. of Genes**	**No. of miRNAs**
Ion binding	7.11 × 10^−65^	995	15
Molecular function	2.91 × 10^−57^	2486	16
RNA binding	1.33 × 10^−50^	427	15
Enzyme binding	8.85 × 10^−45^	302	15
Protein binding transcription factor activity	6.63 × 10^−29^	130	14
poly(A) RNA binding	1.49 × 10^−25^	353	15
Nucleic acid binding transcription factor activity	1.77 × 10^−14^	177	14
**GO Category—Cellular Components**	***p*-Value**	**No. of Genes**	**No. of miRNAs**
Organelle	6.50 × 10^−269^	1890	15
Nucleoplasm	2.07 × 10^−70^	338	13
Protein complex	9.83 × 10^−64^	749	15
Cytosol	1.30 × 10^−56^	574	15
Cellular component	1.75 × 10^−49^	2490	16
Focal adhesion	4.33 × 10^−7^	117	13
**KEGG Pathway**	***p*-Value**	**No. of Genes**	**No. of miRNAs**
Fatty acid biosynthesis	4.74 × 10^−9^	2	3
Adherens junction	2.23 × 10^−6^	29	12
p53 signaling pathway	2.23 × 10^−6^	34	13
Oocyte meiosis	6.46 × 10^−6^	39	10
Cell cycle	1.25 × 10^−5^	46	11
Central carbon metabolism in cancer	1.25 × 10^−5^	27	11
Protein processing in endoplasmic reticulum	1.25 × 10^−5^	60	12
Hippo signaling pathway	1.73 × 10^−5^	46	14
Viral carcinogenesis	1.87 × 10^−5^	59	12

## Data Availability

The datasets analyzed during the current study are available from the corresponding author on reasonable request.

## Supplementary Materials

The following supporting information can be downloaded at: https://www.mdpi.com/article/10.3390/cancers15204910/s1, Serum miRNA-seq analyses: Tables S1–S6 and Figures S1–S15. Figure S1: The analysis workflow for miRNA-seq data; Table S1: A general description of the samples included in the analysis; Table S2: Alignment metrics for spike-in reads. Table S3: Alignment metrics for genomic reads. Figure S2: Histogram representing the distribution of reads in feature counting for samples S1–S25. Figure S3: Histogram representing the distribution of reads in feature counting for samples S26–S50. Figure S4: Histogram representing the distribution of reads in feature counting for samples S51–S75. Figure S5: Histogram representing the distribution of reads in feature counting for samples S76–S100. Figure S6: Histogram representing the distribution of reads in feature counting for samples S101–S125. Table S4: The distribution of the TPM values in each sample. Figure S7: Snapshot from IGV Genome Browser. Figure S8: A curve describing a part of the expression value distribution of the samples in this study. Most genes yield very small expression values and only a few genes have high values. Table S5: GroupWise correlation values for spike-ins. Table S6: GroupWise correlation values for genomic reads. Figure S9: Sample correlations for spike-in reads. Figure S10: Sample correlations for genomic reads. Figure S11: Sample correlations for genomic reads without tissue samples. Figure S12: Hierarchical clustering for spike-in reads. Figure S13: Hierarchical clustering for genomic reads. Figure S14: PCA plot for spike-in reads. Figure S15: PCA plot for genomic read. Table S7: List of 28 upregulated miRNAs in NSCLC vs. control. Table S8: The normalized expression levels of 28 upregulated miRNAs for individual samples. Table S9–14. Table S9: DEmiRNAs between cancerous and non-cancerous patients. Table S10: Shared DE miRNAs between subgroups. Table S11: NSCLC vs. control_GO_BP_results. Table S12: NSCLC vs. control_GO_CC_results. Table S13: NSCLC vs. control_GO_MF_results. Table S14: NSCLC vs. control_KEGG_results; Table S15: The normalized expression levels of 2588 miRNAs for individual samples.
