# Supplementary material for: Serum Insights: Leveraging the Power of miRNA Profiling as an Early Diagnostic Tool for Non-Small Cell Lung Cancer
Source: Cancers (Basel). 2023 Oct 10;15(20):4910. doi: 10.3390/cancers15204910 (PMC10605272; doi:10.3390/cancers15204910)
Supplement: Supplementary file 1 [file cancers-15-04910-s001.zip › Tables S1¿CS6 Figures S1¿CS15_serum miRNA-seq analysis_rev.pdf]

Serum miRNA-seq analysis

Introduction 2

Samples 3

Data preprocessing 6

Aligning reads to the reference genome........... 6

Calculating normalized gene counts........... 17

Files for genome browsers........... 20

Quality control 21

Expression values........... 21

Correlations........... 23

Hierarchical clustering........... 26

PCA........... 28

Differential expression analysis 30

Filtering parameters........... 30

Introduction

The analysis of differential expression from miRNA-seq data consists of several steps and they are presented in the figure below. The initial step in the analysis is aligning the transcriptome sequence reads to the reference genome, followed by counting the number of reads for each miRNA (Section **Data preprocessing**). The quality of the mapping and sample relations are studied using several different methods and visualization techniques in Section **Quality control**. If low quality samples or data outliers are detected they may be excluded from further analysis at this point. The data are also normalized (Section **Data preprocessing**) to reduce systematic noise caused by non-biological sources and to improve the comparability of the samples.

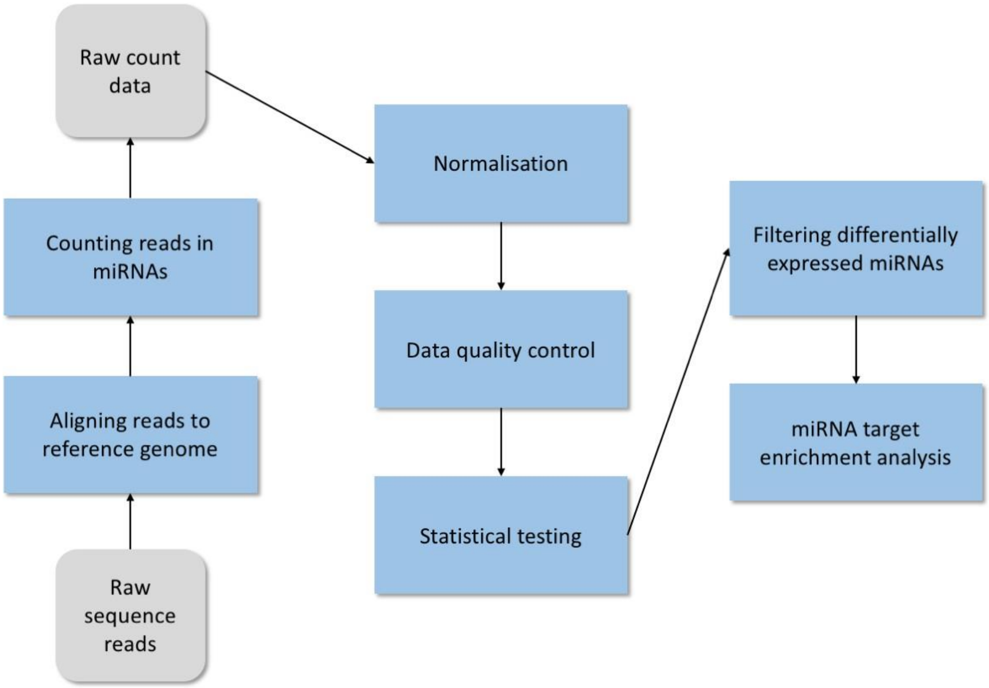

Figure S1: The analysis workflow for miRNA-seq data.

Samples

Table S1. A general description of the samples included in the analysis.

sampleName	groupName	origName
S1	tissue	1Gm_S1_L001_R1_001.fastq.gz
S2	tissue	1N_S2_L001_R1_001.fastq.gz
S3	tissue	2G_S3_L001_R1_001.fastq.gz
S4	tissue	2N_S4_L001_R1_001.fastq.gz
S5	tissue	4Gm_S5_L001_R1_001.fastq.gz
S6	tissue	4N_S6_L001_R1_001.fastq.gz
S7	NSCLC	S66_S7_L001_R1_001.fastq.gZ
S8	tissue	5N_S8_L001_R1_001.fastq.gz
S9	NSCLC	S1_S9_L001_R1_001.fastq.gz
S10	NSCLC	S2_S10_L001_R1_001.fastq.gz
S11	NSCLC	S3_S11_L001_R1_001.fastq.gz
S12	NSCLC	S4_S12_L001_R1_001.fastq.gz
S13	NSCLC	S5_S13_L001_R1_001.fastq.gz
S14	NSCLC	S6_S14_L001_R1_001.fastq.gz
S15	NSCLC	S7_S15_L001_R1_001.fastq.gz
S16	NSCLC	S8_S16_L001_R1_001.fastq.gz
S17	NSCLC	S55_S17_L002_R1_001.fastq.gz
S18	NSCLC	S57_S18_L002_R1_001.fastq.gz
S19	NSCLC	S64_S19_L002_R1_001.fastq.gz
S20	NSCLC	S26_S20_L002_R1_001.fastq.gz
S21	NSCLC	S27_S21_L002_R1_001.fastq.gz
S22	NSCLC	S28_S22_L002_R1_001.fastq.gz
S23	NSCLC	S29_S23_L002_R1_001.fastq.gz

sampleName	groupName	origName
S24	NSCLC	S30_S24_L002_R1_001.fastq.gz
S25	NSCLC	S31_S25_L002_R1_001.fastq.gz
S26	NSCLC	S32_S26_L002_R1_001.fastq.gz
S27	NSCLC	S33_S27_L002_R1_001.fastq.gz
S28	NSCLC	S34_S28_L002_R1_001.fastq.gz
S29	NSCLC	S35_S29_L002_R1_001.fastq.gz
S30	NSCLC	S36_S30_L002_R1_001.fastq.gz
S31	NSCLC	S39_S31_L002_R1_001.fastq.gz
S32	NSCLC	S40_S32_L002_R1_001.fastq.gz
S33	NSCLC	S59_S33_L003_R1_001.fastq.gz
S34	NSCLC	S68_S34_L003_R1_001.fastq.gz
S35	NSCLC	S15_S35_L003_R1_001.fastq.gz
S36	NSCLC	S16_S36_L003_R1_001.fastq.gz
S37	NSCLC	S17_S37_L003_R1_001.fastq.gz
S38	NSCLC	S18_S38_L003_R1_001.fastq.gz
S39	NSCLC	S19_S39_L003_R1_001.fastq.gz
S40	NSCLC	S20_S40_L003_R1_001.fastq.gz
S41	NSCLC	S21_S41_L003_R1_001.fastq.gZ
S42	NSCLC	S22_S42_L003_R1_001.fastq.gz
S43	NSCLC	S23_S43_L003_R1_001.fastq.gz
S44	NSCLC	S12_S44_L003_R1_001.fastq.gz
S45	NSCLC	S24_S45_L003_R1_001.fastq.gz
S46	NSCLC	S25_S46_L003_R1_001.fastq.gz
S47	NSCLC	S37_S47_L003_R1_001.fastq.gz
S48	NSCLC	S38_S48_L003_R1_001.fastq.gz
S49	NSCLC	S53_S49_L004_R1_001.fastq.gz
S50	NSCLC	S62_S50_L004_R1_001.fastq.gz
S51	NSCLC	S67_S51_L004_R1_001.fastq.gz
S52	NSCLC	S48_S52_L004_R1_001.fastq.gz
S53	NSCLC	S71_S53_L004_R1_001.fastq.gz
S54	Control	S72_S54_L004_R1_001.fastq.gz
S55	Control	S73_S55_L004_R1_001.fastq.gz
S56	Control	S74_S56_L004_R1_001.fastq.gz
S57	Control	S75_S57_L004_R1_001.fastq.gz
S58	Control	S76_S58_L004_R1_001. fastq.gz
S59	Control	S77_S59_L004_R1_001.fastq.gz
S60	Control	S78_S60_L004_R1_001.fastq.gz
S61	Control	S79_S61_L004_R1_001.fastq.gz
S62	Control	S80_S62_L004_R1_001.fastq.gz
S63	Control	S81_S63_L004_R1_001.fastq.gz
S64	Control	S82_S64_L004_R1_001.fastq.gz
S65	NSCLC	S54_S65_L005_R1_001.fastq.gz
S66	NSCLC	S63_S66_L005_R1_001.fastq.gz
S67	Control	S85_S67_L005_R1_001.fastq.gz
S68	NSCLC	S9_S68_L005_R1_001.fastq.gz
S69	NSCLC	S10_S69_L005_R1_001.fastq.gz
S70	NSCLC	S11_S70_L005_R1_001.fastq.gz
S71	Control	S86_S71_L005_R1_001.fastq.gz
S72	Control	S87_S72_L005_R1_001.fastq.gz
S73	Control	S88_S73_L005_R1_001.fastq.gz
S74	Control	S89_S74_L005_R1_001.fastq.gz
S75	Control	S90_S75_L005_R1_001.fastq.gz

sampleName	groupName	origName
S76	Control	S91_S76_L005_R1_001.fastq.gz
S77	Control	S92_S77_L005_R1_001.fastq.gz
S78	Control	S93_S78_L005_R1_001.fastq.gz
S79	Control	S94_S79_L005_R1_001.fastq.gz
S80	Control	S95_S80_L005_R1_001.fastq.gz
S81	NSCLC	S60_S81_L006_R1_001.fastq.gz
S82	NSCLC	S69_S82_L006_R1_001.fastq.gz
S83	NSCLC	S13_S83_L006_R1_001.fastq.gz
S84	NSCLC	S14_S84_L006_R1_001.fastq.gz
S85	Control	S96_S85_L006_R1_001.fastq.gz
S86	Control	S97_S86_L006_R1_001.fastq.gz
S87	Control	S98_S87_L006_R1_001.fastq.gz
S88	Control	S99_S88_L006_R1_001.fastq.gz
S89	Control	S100_S89_L006_R1_001.fastq.gz
S90	Control	S101_S90_L006_R1_001.fastq.gz
S91	Control	S102_S91_L006_R1_001.fastq.gz
S92	Control	S103_S92_L006_R1_001.fastq.gz
S93	Control	S104_S93_L006_R1_001.fastq.gz
S94	Control	S105_S94_L006_R1_001.fastq.gz
S95	Control	S106_S95_L006_R1_001.fastq.gz
S96	Control	S107_S96_L006_R1_001.fastq.gz
S97	NSCLC	S56_S97_L007_R1_001.fastq.gz
S98	NSCLC	S58_S98_L007_R1_001.fastq.gz
S99	NSCLC	S65_S99_L007_R1_001.fastq.gz
S100	NSCLC	S41_S100_L007_R1_001.fastq.gz
S101	NSCLC	S42_S101_L007_R1_001.fastq.gz
S102	NSCLC	S43_S102_L007_R1_001.fastq.gz
S103	NSCLC	S44_S103_L007_R1_001.fastq.gz
S104	NSCLC	S45_S104_L007_R1_001.fastq.gz
S105	NSCLC	S46_S105_L007_R1_001.fastq.gz
S106	NSCLC	S47_S106_L007_R1_001.fastq.gz
S107	NSCLC	S49_S107_L007_R1_001.fastq.gz
S108	NSCLC	S50_S108_L007_R1_001.fastq.gz
S109	NSCLC	S51_S109_L007_R1_001.fastq.gz
S110	NSCLC	S52_S110_L007_R1_001.fastq.gz
S111	Control	S83_S111_L007_R1_001.fastq.gz
S112	Control	S84_S112_L007_R1_001.fastq.gz
S113	NSCLC	S61_S113_L008_R1_001.fastq.gz
S114	NSCLC	S70_S114_L008_R1_001.fastq.gz
S115	Control	S108_S115_L008_R1_001.fastq.gz
S116	Control	S109_S116_L008_R1_001.fastq.gz
S117	Control	S111_S117_L008_R1_001.fastq.gz
S118	Control	S112_S118_L008_R1_001.fastq.gz
S119	Control	S113_S119_L008_R1_001.fastq.gz
S120	Control	S114_S120_L008_R1_001.fastq.gz
S121	Control	S115_S121_L008_R1_001.fastq.gz
S122	Control	S116_S122_L008_R1_001.fastq.gz
S123	Control	S117_S123_L008_R1_001.fastq.gz
S124	Control	S118_S124_L008_R1_001.fastq.gz
S125	Control	S119_S125_L008_R1_001.fastq.gz

Data preprocessing

Aligning reads to the reference genome

The reads obtained from the instrument were base called using the instrument manufacturer's base calling software. Prior to alignment the reads were trimmed with cutadapt first to remove the adapter contamination and then to trim the four random bases from both ends of the reads.

The samples contained ExiSEQ spike-ins, so first the reads were aligned to the spike-ins with Bowtie2. The percentage of reads mapping to the spike-ins is provided in the table below.

Samples S1, S2, S3, S4, S5, S6, and S8 originated from lung tumors and were not part of the current study.

Table S2: Alignment metrics for spike-in reads

Sample	Total reads	Uniquely mapped	% uniquely mapped	Multiply mapped	% multiply mapped
S1_tissue	15723608	99745	0.63%	23	0.00%
S2_tissue	28017692	323239	1.15%	28	0.00%
S3_tissue	21280830	268931	1.26%	13	0.00%
S4_tissue	20491251	191558	0.93%	25	0.00%
S5_tissue	17683215	147610	0.83%	20	0.00%
S6_tissue	24134335	404744	1.68%	69	0.00%
S7_NSCLC	21763760	316345	1.45%	2	0.00%
S8_tissue	25153442	301268	1.20%	8	0.00%
S9_NSCLC	12921140	266650	2.06%	196	0.00%
S10_NSCLC	22887717	233556	1.02%	329	0.00%
S11_NSCLC	25516327	319044	1.25%	0	0.00%
S12_NSCLC	20415989	134283	0.66%	2	0.00%
S13_NSCLC	18606846	302775	1.63%	374	0.00%
S14_NSCLC	20689020	418230	2.02%	251	0.00%
S15_NSCLC	18777929	225196	1.20%	55	0.00%
S16_NSCLC	20884435	475427	2.28%	673	0.00%
S17_NSCLC	18318017	14215	0.08%	1	0.00%
S18_NSCLC	15809607	89994	0.57%	77	0.00%
S19_NSCLC	14914307	39620	0.27%	0	0.00%
S20_NSCLC	12152018	273032	2.25%	226	0.00%
S21_NSCLC	12993984	178153	1.37%	113	0.00%
S22_NSCLC	15323543	349261	2.28%	486	0.00%
S23_NSCLC	20839135	172716	0.83%	1	0.00%
S24_NSCLC	15363302	128268	0.83%	2	0.00%
S25_NSCLC	15016833	365808	2.44%	386	0.00%
S26_NSCLC	15092710	418187	2.77%	230	0.00%
S27_NSCLC	14790432	393278	2.66%	531	0.00%
S28_NSCLC	13528748	391941	2.90%	275	0.00%
S29_NSCLC	17993628	292502	1.63%	786	0.00%
S30_NSCLC	15209731	228180	1.50%	308	0.00%
S31_NSCLC	15857805	5049	0.03%	0	0.00%
S32_NSCLC	12111478	305783	2.52%	400	0.00%
S33_NSCLC	23272221	94179	0.40%	1	0.00%
S34_NSCLC	20230213	123405	0.61%	3	0.00%
S35_NSCLC	19797821	464935	2.35%	250	0.00%
S36_NSCLC	17648154	876354	4.97%	880	0.00%
S37_NSCLC	18981445	375029	1.98%	408	0.00%
S38_NSCLC	20705938	326286	1.58%	563	0.00%
S39_NSCLC	16399461	367774	2.24%	386	0.00%

S40_NSCLC	25463960	385451	1.51%	505	0.00%

Sample	Total reads	Uniquely mapped	% uniquely mapped	Multiply mapped	% multiply mapped
S41_NSCLC	17343461	427536	2.47%	1104	0.01%
S42_NSCLC	19248447	492784	2.56%	462	0.00%
S43_NSCLC	21093707	174837	0.83%	0	0.00%
S44_NSCLC	16278765	46639	0.29%	1435	0.01%
S45_NSCLC	14783046	8237	0.06%	0	0.00%
S46_NSCLC	20391890	23100	0.11%	1	0.00%
S47_NSCLC	20389118	13943	0.07%	0	0.00%
S48_NSCLC	18004481	17262	0.10%	1	0.00%
S49_NSCLC	22874462	49062	0.21%	0	0.00%
S50_NSCLC	16897339	106036	0.63%	0	0.00%
S51_NSCLC	25134011	72888	0.29%	6	0.00%
S52_NSCLC	21872797	75504	0.35%	0	0.00%
S53_NSCLC	22919205	463838	2.02%	350	0.00%
S54_Control	13643738	292606	2.14%	297	0.00%
S55_Control	16925216	244681	1.45%	321	0.00%
S56_Control	18874921	518240	2.75%	320	0.00%
S57_Control	11599851	249584	2.15%	260	0.00%
S58_Control	13229780	324787	2.45%	509	0.00%
S59_Control	22676456	584585	2.58%	588	0.00%
S60_Control	15999352	489614	3.06%	500	0.00%
S61_Control	20961032	361021	1.72%	118	0.00%
S62_Control	18173181	229047	1.26%	1	0.00%
S63_Control	20969901	262483	1.25%	208	0.00%
S64_Control	18954578	325848	1.72%	188	0.00%
S65_NSCLC	22220830	30476	0.14%	0	0.00%
S66_NSCLC	15542265	154850	1.00%	4	0.00%
S67_Control	19303144	249643	1.29%	1	0.00%
S68_NSCLC	18036408	274626	1.52%	390	0.00%
S69_NSCLC	14255076	429908	3.02%	844	0.01%
S70_NSCLC	16045186	550296	3.43%	818	0.01%
S71_Control	15769310	425953	2.70%	151	0.00%
S72_Control	16792542	429867	2.56%	604	0.00%
S73_Control	20001245	326458	1.63%	219	0.00%
S74_Control	23441576	644912	2.75%	668	0.00%
S75_Control	20084322	473423	2.36%	968	0.00%
S76_Control	22153908	675252	3.05%	673	0.00%
S77_Control	21232876	739848	3.48%	715	0.00%
S78_Control	23072030	367102	1.59%	541	0.00%
S79_Control	20097737	537631	2.68%	371	0.00%
S80_Control	29190164	830102	2.84%	1052	0.00%
S81_NSCLC	23428444	329692	1.41%	216	0.00%
S82_NSCLC	16671912	44118	0.26%	0	0.00%
S83_NSCLC	20846268	1131749	5.43%	950	0.00%
S84_NSCLC	19247191	445170	2.31%	444	0.00%
S85_Control	20468848	631864	3.09%	1244	0.01%
S86_Control	15959776	510916	3.20%	588	0.00%
S87_Control	18429513	499459	2.71%	1190	0.01%
S88_Control	21280015	479320	2.25%	3	0.00%
S89_Control	20224136	696320	3.44%	722	0.00%
S90_Control	24810610	527783	2.13%	849	0.00%
S91_Control	21241871	463606	2.18%	1989	0.01%
S92_Control	20033562	675940	3.37%	575	0.00%

Sample	Total reads	Uniquely mapped	% uniquely mapped	Multiply mapped	% multiply mapped
S93_Control	17130268	315933	1.84%	372	0.00%
S94_Control	12448685	443956	3.57%	279	0.00%
S95_Control	20635636	210430	1.02%	6	0.00%
S96_Control	23710691	345430	1.46%	281	0.00%
S97_NSCLC	23667586	104719	0.44%	0	0.00%
S98_NSCLC	21532696	115422	0.54%	4	0.00%
S99_NSCLC	19336124	28529	0.15%	0	0.00%
S100_NSCLC	16724289	12009	0.07%	0	0.00%
S101_NSCLC	17290180	151203	0.87%	0	0.00%
S102_NSCLC	17713070	94065	0.53%	0	0.00%
S103_NSCLC	17421167	72427	0.42%	0	0.00%
S104_NSCLC	17203235	12336	0.07%	0	0.00%
S105_NSCLC	18469163	58092	0.31%	0	0.00%
S106_NSCLC	24504236	100257	0.41%	35	0.00%
S107_NSCLC	25885897	83561	0.32%	0	0.00%
S108_NSCLC	19912881	20796	0.10%	0	0.00%
S109_NSCLC	19632303	12055	0.06%	64	0.00%
S110_NSCLC	19115945	36157	0.19%	2	0.00%
S111_Control	18222655	344070	1.89%	464	0.00%
S112_Control	17496531	201909	1.15%	0	0.00%
S113_NSCLC	19315162	341191	1.77%	211	0.00%
S114_NSCLC	21359010	578940	2.71%	823	0.00%
S115_Control	28397481	392940	1.38%	523	0.00%
S116_Control	23524507	577182	2.45%	910	0.00%
S117_Control	23025338	1049941	4.56%	381	0.00%
S118_Control	26710149	330671	1.24%	1	0.00%
S119_Control	23627962	536871	2.27%	2	0.00%
S120_Control	19064047	497021	2.61%	220	0.00%
S121_Control	27268667	720990	2.64%	1734	0.01%
S122_Control	23522916	251949	1.07%	169	0.00%
S123_Control	20982578	207506	0.99%	430	0.00%
S124_Control	17661138	25124	0.14%	2	0.00%
S125_Control	19438159	204292	1.05%	3	0.00%

The remaining reads were then aligned against the *Homo sapiens* reference genome (Ensembl GRCh38 release) with STAR version 2.5.3a using 2-pass alignment mode. The miRbase annotation was used in both mapping and read counting. The mapping percentages varied between the samples, and a summary of the alignment statistics is provided in the table below.

Table S3: Alignment metrics for genomic reads

Sample	Reads	Unique mapped	% unique mapped	Multiple mapped	% multiple mapped
S1_tissue	15723608	12531494	79.70%	2852462	18.14%
S2_tissue	28017692	17315433	61.80%	8055588	28.75%
S3_tissue	21280830	13960508	65.60%	5258867	24.71%
S4_tissue	20491251	14774426	72.10%	5352261	26.12%
S5_tissue	17683215	12363155	69.91%	4799998	27.14%
S6_tissue	24134335	16428667	68.07%	7121163	29.51%
S7_NSCLC	21763760	6304303	28.97%	10739014	49.34%
S8_tissue	25153442	16217808	64.48%	7677914	30.52%
S9_NSCLC	12921140	5369512	41.56%	5506236	42.61%

Sample	Reads	Unique mapped	% unique mapped	Multiple mapped	% multiple mapped
S10_NSCLC	22887717	9922876	43.35%	8465153	36.99%
S11_NSCLC	25516327	8403385	32.93%	10499139	41.15%
S12_NSCLC	20415989	5410710	26.50%	8801471	43.11%
S13_NSCLC	18606846	7964698	42.81%	7743905	41.62%
S14_NSCLC	20689020	6918329	33.44%	8769830	42.39%
S15_NSCLC	18777929	6099979	32.48%	7683331	40.92%
S16_NSCLC	20884435	7237139	34.65%	9926978	47.53%
S17_NSCLC	18318017	4339149	23.69%	8711621	47.56%
S18_NSCLC	15809607	4895297	30.96%	6990677	44.22%
S19_NSCLC	14914307	3943591	26.44%	6854549	45.96%
S20_NSCLC	12152018	4753042	39.11%	5139301	42.29%
S21_NSCLC	12993984	5041527	38.80%	4627092	35.61%
S22_NSCLC	15323543	6625329	43.24%	6640115	43.33%
S23_NSCLC	20839135	5121534	24.58%	7345596	35.25%
S24_NSCLC	15363302	4688936	30.52%	7119118	46.34%
S25_NSCLC	15016833	7309217	48.67%	5492322	36.57%
S26_NSCLC	15092710	5539610	36.70%	6029442	39.95%
S27_NSCLC	14790432	5835853	39.46%	5800867	39.22%
S28_NSCLC	13528748	4967788	36.72%	5840583	43.17%
S29_NSCLC	17993628	6576243	36.55%	7504669	41.71%
S30_NSCLC	15209731	5829551	38.33%	6406123	42.12%
S31_NSCLC	15857805	4519378	28.50%	7015384	44.24%
S32_NSCLC	12111478	4967947	41.02%	4927120	40.68%
S33_NSCLC	23272221	6513740	27.99%	11584983	49.78%
S34_NSCLC	20230213	5870503	29.02%	9349114	46.21%
S35_NSCLC	19797821	7402037	37.39%	7075917	35.74%
S36_NSCLC	17648154	7764871	44.00%	7160994	40.58%
S37_NSCLC	18981445	8048611	42.40%	7643709	40.27%
S38_NSCLC	20705938	7417170	35.82%	8672259	41.88%
S39_NSCLC	16399461	7002055	42.70%	6552837	39.96%
S40_NSCLC	25463960	12367974	48.57%	8379779	32.91%
S41_NSCLC	17343461	7021383	40.48%	7102104	40.95%
S42_NSCLC	19248447	8352904	43.40%	8033567	41.74%
S43_NSCLC	21093707	10428589	49.44%	7422832	35.19%
S44_NSCLC	16278765	4248686	26.10%	7649426	46.99%
S45_NSCLC	14783046	4350807	29.43%	6896817	46.65%
S46_NSCLC	20391890	6692240	32.82%	8933474	43.81%
S47_NSCLC	20389118	5816870	28.53%	9480994	46.50%
S48_NSCLC	18004481	5550503	30.83%	7903082	43.90%
S49_NSCLC	22874462	4979913	21.77%	10496542	45.89%
S50_NSCLC	16897339	4130409	24.44%	8176511	48.39%
S51_NSCLC	25134011	5541965	22.05%	13505514	53.73%
S52_NSCLC	21872797	5849719	26.74%	10561686	48.29%
S53_NSCLC	22919205	8826876	38.51%	8820836	38.49%
S54_Control	13643738	5570593	40.83%	5598067	41.03%
S55_Control	16925216	7000184	41.36%	6731989	39.77%
S56_Control	18874921	7380097	39.10%	8105931	42.95%
S57_Control	11599851	4173819	35.98%	4653838	40.12%
S58_Control	13229780	5386647	40.72%	5293139	40.01%
S59_Control	22676456	10345999	45.62%	9156553	40.38%
S60_Control	15999352	6492144	40.58%	6773744	42.34%
S61_Control	20961032	6996452	33.38%	7479351	35.68%

Sample	Reads	Unique mapped	% unique mapped	Multiple mapped	% multiple mapped
S62_Control	18173181	7011347	38.58%	7034979	38.71%
S63_Control	20969901	7143202	34.06%	9178993	43.77%
S64_Control	18954578	7412977	39.11%	6837187	36.07%
S65_NSCLC	22220830	6221160	28.00%	10410228	46.85%
S66_NSCLC	15542265	4202939	27.04%	8313593	53.49%
S67_Control	19303144	6512061	33.74%	8038669	41.64%
S68_NSCLC	18036408	6340734	35.16%	7116844	39.46%
S69_NSCLC	14255076	6297334	44.18%	5871579	41.19%
S70_NSCLC	16045186	6812710	42.46%	6467998	40.31%
S71_Control	15769310	6057446	38.41%	6170267	39.13%
S72_Control	16792542	5728486	34.11%	7780675	46.33%
S73_Control	20001245	5493722	27.47%	8908304	44.54%
S74_Control	23441576	8595946	36.67%	9591325	40.92%
S75_Control	20084322	6617799	32.95%	8480471	42.22%
S76_Control	22153908	7691040	34.72%	10254917	46.29%
S77_Control	21232876	7936897	37.38%	9458035	44.54%
S78_Control	23072030	7742605	33.56%	9438380	40.91%
S79_Control	20097737	8552604	42.56%	7543776	37.54%
S80_Control	29190164	12135066	41.57%	11290369	38.68%
S81_NSCLC	23428444	6381732	27.24%	10772993	45.98%
S82_NSCLC	16671912	4566511	27.39%	8074537	48.43%
S83_NSCLC	20846268	8179710	39.24%	8610167	41.30%
S84_NSCLC	19247191	8882143	46.15%	6224346	32.34%
S85_Control	20468848	8128054	39.71%	7871547	38.46%
S86_Control	15959776	6068020	38.02%	7222988	45.26%
S87_Control	18429513	6752035	36.64%	7727423	41.93%
S88_Control	21280015	6542904	30.75%	9320514	43.80%
S89_Control	20224136	7614633	37.65%	8780886	43.42%
S90_Control	24810610	9283930	37.42%	8994433	36.25%
S91_Control	21241871	7603342	35.79%	8543346	40.22%
S92_Control	20033562	7242989	36.15%	8702010	43.44%
S93_Control	17130268	5148246	30.05%	7794974	45.50%
S94_Control	12448685	5361902	43.07%	5125173	41.17%
S95_Control	20635636	6296213	30.51%	8365253	40.54%
S96_Control	23710691	8046408	33.94%	7530414	31.76%
S97_NSCLC	23667586	5805781	24.53%	11621827	49.10%
S98_NSCLC	21532696	6255992	29.05%	9695149	45.03%
S99_NSCLC	19336124	6504760	33.64%	8494585	43.93%
S100_NSCLC	16724289	4934509	29.51%	7561276	45.21%
S101_NSCLC	17290180	5500006	31.81%	9293397	53.75%
S102_NSCLC	17713070	5322455	30.05%	7996800	45.15%
S103_NSCLC	17421167	5046625	28.97%	8091363	46.45%
S104_NSCLC	17203235	5111295	29.71%	7581176	44.07%
S105_NSCLC	18469163	5454480	29.53%	8102729	43.87%
S106_NSCLC	24504236	7651642	31.23%	9286308	37.90%
S107_NSCLC	25885897	7169515	27.70%	11815390	45.64%
S108_NSCLC	19912881	6636664	33.33%	7896704	39.66%
S109_NSCLC	19632303	5340168	27.20%	8832472	44.99%
S110_NSCLC	19115945	5951769	31.14%	8420761	44.05%
S111_Control	18222655	7318109	40.16%	7737399	42.46%
S112_Control	17496531	6138799	35.09%	8024056	45.86%
S113_NSCLC	19315162	6283527	32.53%	9131125	47.27%

Sample	Reads	Unique mapped	% unique mapped	Multiple mapped	% multiple mapped
S114_NSCLC	21359010	8002730	37.47%	9134030	42.76%
S115_Control	28397481	10216966	35.98%	11939622	42.04%
S116_Control	23524507	9272876	39.42%	9764762	41.51%
S117_Control	23025338	9178295	39.86%	8973421	38.97%
S118_Control	26710149	11245221	42.10%	9916365	37.13%
S119_Control	23627962	9085531	38.45%	10303243	43.61%
S120_Control	19064047	7744792	40.63%	7945900	41.68%
S121_Control	27268667	10522094	38.59%	11740908	43.06%
S122_Control	23522916	9796369	41.65%	9438056	40.12%
S123_Control	20982578	6953202	33.14%	8911537	42.47%
S124_Control	17661138	4848786	27.45%	8308865	47.05%
S125_Control	19438159	6638318	34.15%	8614028	44.32%

After alignment, the number of both spike-ins and genomic reads assigned to each miRNA was counted using featureCounts from R package Rsubread. For genomic reads multiply mapping reads were also taken into account. All (100%) of the spike-in reads could be assigned to the spike-ins. The histograms show the distribution of the genomic read counts after featureCounts analysis. Most of the reads should be assigned to miRNA features.

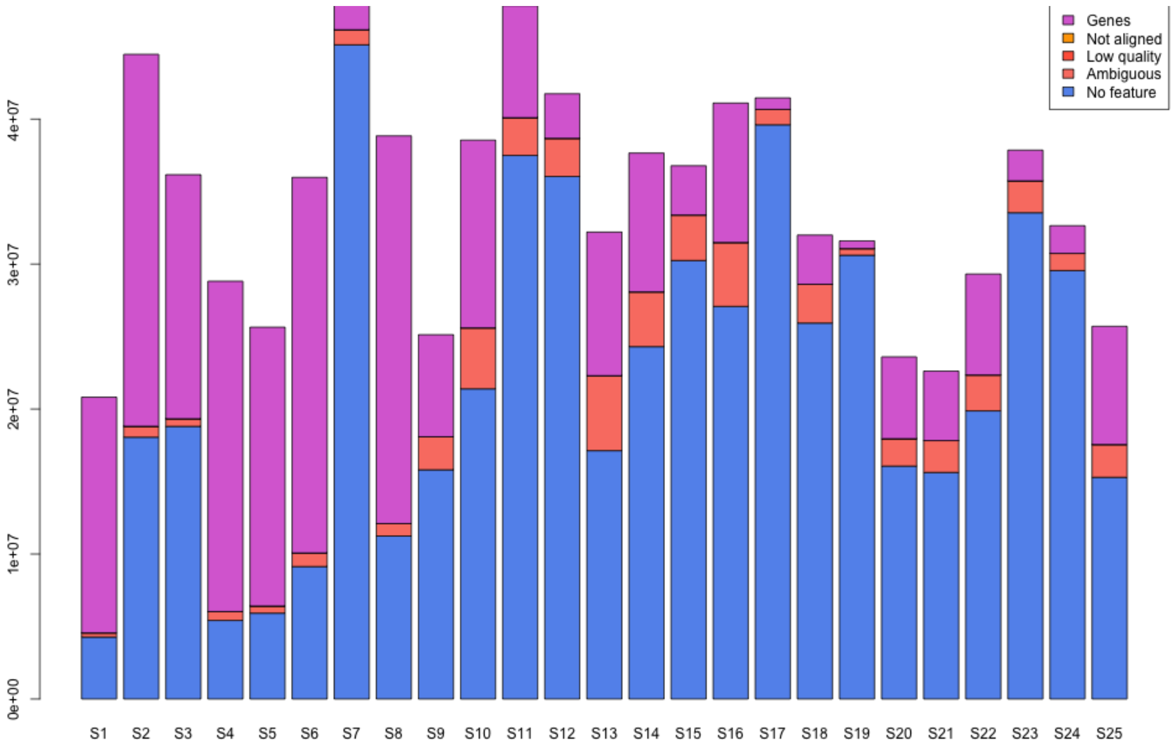

Figure S2: Histogram representing the distribution of reads in feature counting for samples S1-S25.

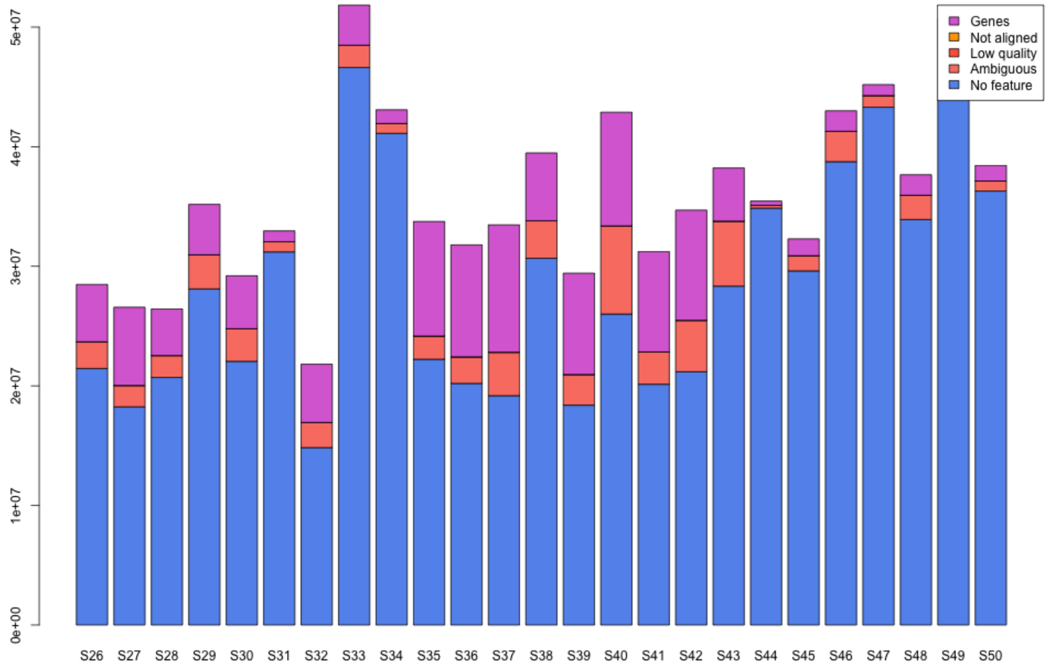

Figure S3: Histogram representing the distribution of reads in feature counting for samples S26-S50.

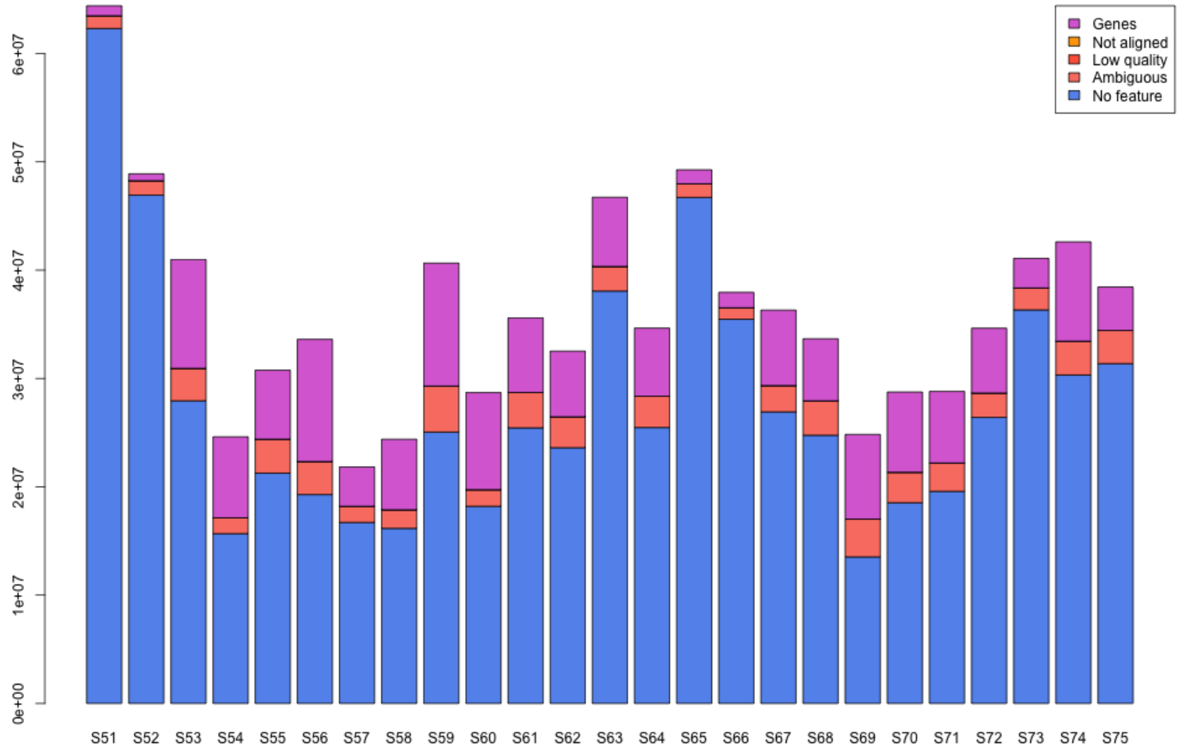

Figure S4: Histogram representing the distribution of reads in feature counting for samples S51-S75.

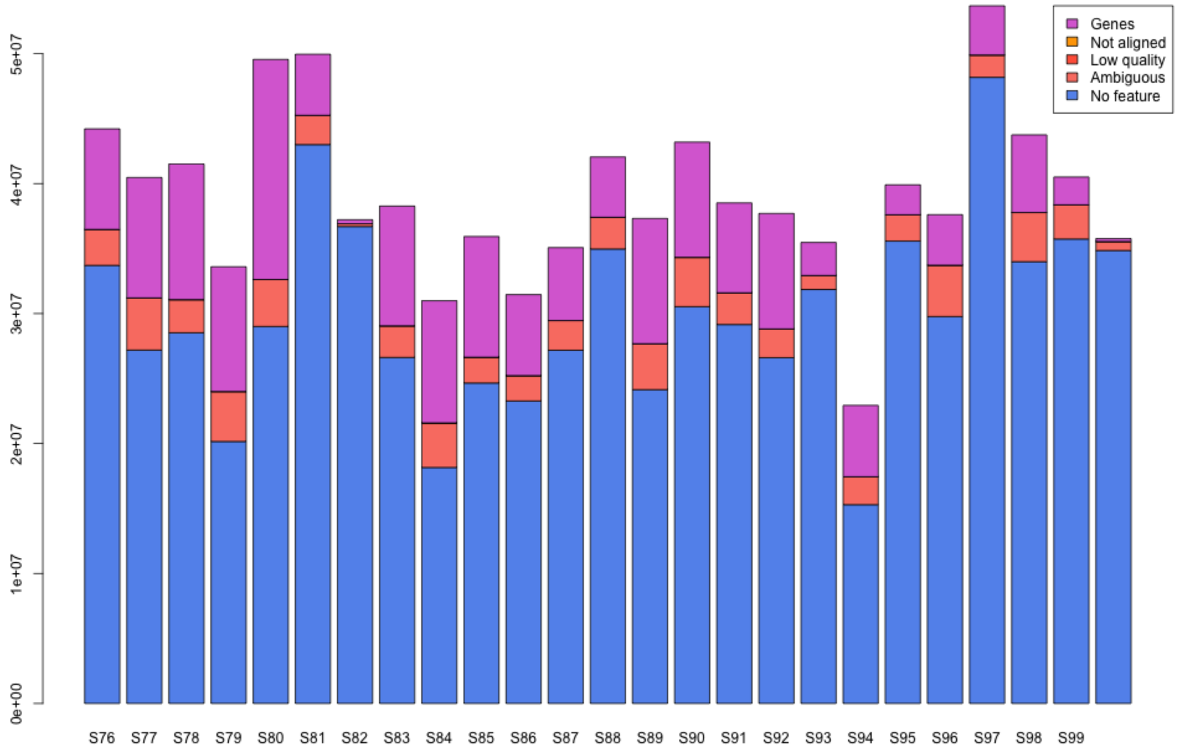

Figure S5: Histogram representing the distribution of reads in feature counting for samples S76-S100.

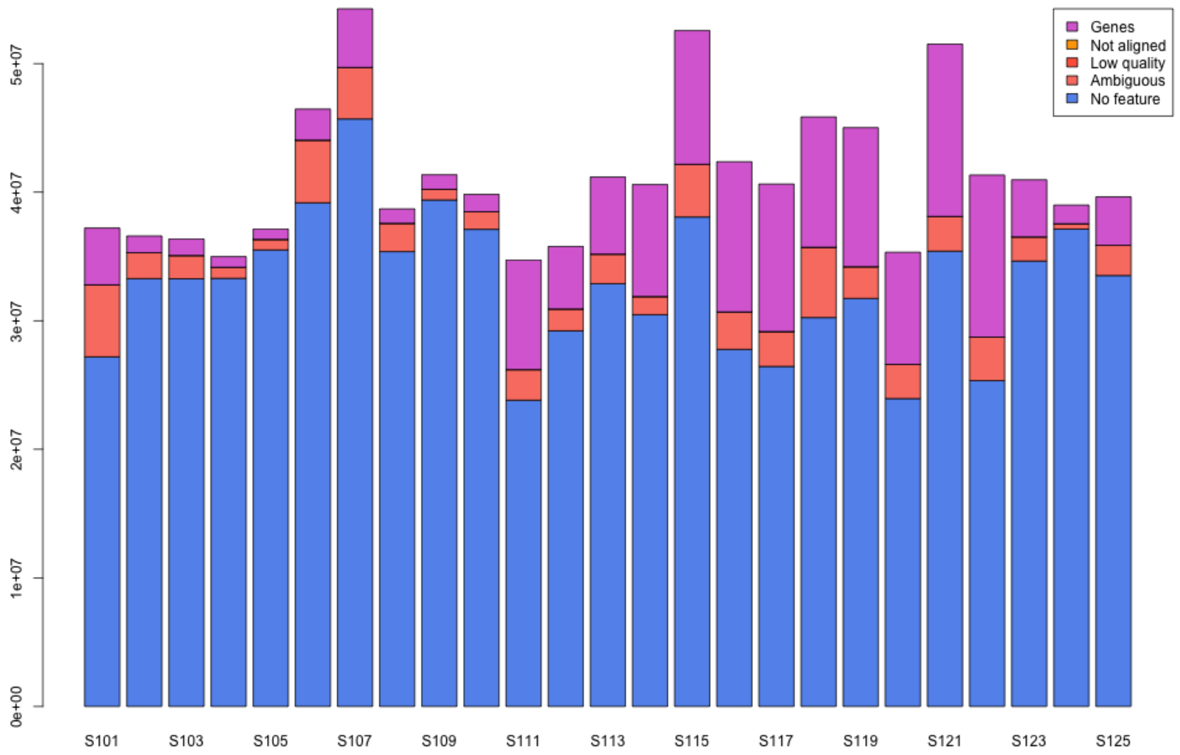

Figure S6: Histogram representing the distribution of reads in feature counting for samples S101-S125.

Calculating normalized gene counts

The data are normalised to remove variation between samples caused by non-biological reasons and to make the values comparable across the sample set. Here the counts were normalised using the TMM normalisation method of the edgeRR/Bioconductor package (R version 3.4.1, Bioconductor version 3.5). The method takes the variable number of total reads across samples into account by calculating specific scaling factors between the samples.

For statistical testing the data were further log transformed using the voom approach in the limma package. For the visualizations and result files the TMM normalised counts are represented as TPM values, which make the values not only between samples but also between genes comparable. However, due to the relative nature of miRNA-seq experiments, we strongly recommend using these values only as approximate measures of expression and not as accurate values, e.g. a gene can beconsidered as lowly, middle or highly expressed based on its TPM value.

The distribution of the TPM values in each sample is presented in the table below. The TPM value column denotes the TPM value threshold and the number of genes that have a TPM value greater than the threshold is given for each sample.

Table S4. The distribution of the TPM values in each sample

TPM value	S1	S2	S3	S4	S5	S6	S7	S8	S9	S10	S11	S12	S13	S14
0	2588	2588	2588	2588	2588	2588	2588	2588	2588	2588	2588	2588	2588	2588
1	505	522	549	507	513	555	327	542	458	462	247	265	347	363
3	355	400	411	378	392	415	260	399	439	446	221	239	341	353
5	325	356	370	337	355	360	230	347	412	426	216	230	338	347
10	264	300	312	279	297	297	219	293	351	350	211	218	302	327
15	233	270	273	252	268	264	211	265	309	296	203	217	274	290
25	204	231	235	215	227	231	207	220	253	264	199	212	220	243
30	195	223	218	207	209	217	206	207	243	247	196	212	203	232
40	182	202	204	186	198	198	202	193	223	227	196	209	186	206
50	171	188	195	174	187	182	199	182	203	207	193	208	173	188
100	131	149	148	139	149	151	189	145	157	159	157	177	131	143

TPM value	S15	S16	S17	S18	S19	S20	S21	S22	S23	S24	S25	S26	S27	S28
0	2588	2588	2588	2588	2588	2588	2588	2588	2588	2588	2588	2588	2588	2588
1	307	295	274	282	285	511	444	454	321	226	425	429	343	354
3	281	288	189	274	205	484	433	436	309	199	412	411	329	346
5	274	282	181	270	180	439	410	407	306	194	384	402	322	343
10	270	271	158	267	159	352	344	344	300	191	321	349	301	335
15	268	259	150	259	146	307	298	303	293	190	270	312	276	315
25	262	228	142	245	135	266	244	256	279	186	224	253	239	281
30	254	211	139	235	134	257	226	236	271	184	212	238	224	267
40	244	185	138	217	132	225	202	214	247	182	190	217	205	234
50	229	173	137	205	130	210	183	192	224	177	175	196	183	213
100	182	136	135	159	125	167	155	150	170	159	134	156	141	170

TPM value	S29	S30	S31	S32	S33	S34	S35	S36	S37	S38	S39	S40	S41	S42
0	2588	2588	2588	2588	2588	2588	2588	2588	2588	2588	2588	2588	2588	2588
1	301	320	237	304	191	244	497	334	476	294	410	413	281	433
3	294	310	157	299	172	206	436	324	450	288	406	408	276	426
5	288	307	131	292	167	189	374	321	397	285	392	390	274	402
10	283	291	115	278	161	180	296	293	318	280	336	356	265	341

TPM value	S29	S30	S31	S32	S33	S34	S35	S36	S37	S38	S39	S40	S41	S42
15	279	280	109	257	161	174	265	272	276	271	302	310	259	293
25	269	259	101	226	161	170	218	221	233	239	255	259	229	239
30	263	254	98	214	161	169	207	210	216	227	241	234	213	226
40	243	231	96	194	161	169	190	192	195	214	218	215	195	206
50	226	209	96	178	160	167	174	177	183	191	194	207	179	189
100	182	159	89	140	154	164	150	146	153	146	152	157	145	156

TPM value	S43	S44	S45	S46	S47	S48	S49	S50	S51	S52	S53	S54	S55	S56
0	2588	2588	2588	2588	2588	2588	2588	2588	2588	2588	2588	2588	2588	2588
1	181	254	189	199	256	200	300	242	362	292	348	291	300	404
3	171	183	152	147	159	141	205	216	237	228	342	285	299	393
5	168	183	143	135	134	128	198	208	213	182	339	279	298	364
10	166	136	129	119	108	110	183	206	184	165	310	271	285	299
15	166	112	127	114	99	105	180	205	182	157	268	258	270	259
25	162	100	124	114	89	101	171	203	174	146	228	232	240	222
30	160	96	124	114	84	101	171	203	174	145	209	219	229	208
40	160	88	123	114	82	97	167	202	174	143	189	205	207	190
50	160	82	122	109	80	96	166	199	172	142	171	196	193	174
100	146	69	113	106	72	93	159	183	166	138	141	154	152	141

TPM value	S57	S58	S59	S60	S61	S62	S63	S64	S65	S66	S67	S68	S69	S70
0	2588	2588	2588	2588	2588	2588	2588	2588	2588	2588	2588	2588	2588	2588
1	309	350	340	314	464	208	322	336	224	284	231	335	421	319
3	301	343	337	313	443	205	319	329	184	258	220	331	411	313
5	299	340	332	310	418	205	314	326	164	249	220	326	397	312
10	294	325	305	299	351	202	304	302	151	247	217	315	334	308
15	280	304	268	274	306	202	286	273	141	245	215	296	298	304
25	264	254	225	235	251	199	247	243	136	242	211	263	229	260
30	253	237	212	220	235	195	231	218	136	241	205	247	218	248
40	229	214	188	203	214	192	209	205	135	237	195	224	201	232
50	209	198	178	193	197	185	194	188	132	225	174	209	190	210
100	152	145	136	150	153	154	149	140	124	195	128	166	148	158

TPM value	S71	S72	S73	S74	S75	S76	S77	S78	S79	S80	S81	S82	S83	S84
0	2588	2588	2588	2588	2588	2588	2588	2588	2588	2588	2588	2588	2588	2588
1	422	338	233	282	252	280	285	274	338	392	355	271	393	561
3	413	329	216	279	246	277	282	270	334	384	343	271	386	489
5	397	324	214	278	243	277	279	268	324	369	342	202	378	411
10	343	305	212	270	239	268	272	263	307	299	329	149	336	338
15	292	279	211	261	238	261	268	254	272	249	314	140	299	296
25	242	243	207	239	232	243	235	211	230	219	274	129	237	241
30	227	232	207	224	228	231	225	200	218	206	264	121	221	228
40	209	209	204	209	219	212	207	177	200	179	233	113	206	210
50	188	198	202	191	207	201	190	165	185	169	214	111	193	191
100	157	145	181	148	155	159	161	131	150	132	163	100	145	154

TPM value	S85	S86	S87	S88	S89	S90	S91	S92	S93	S94	S95	S96	S97	S98
0	2588	2588	2588	2588	2588	2588	2588	2588	2588	2588	2588	2588	2588	2588
1	315	242	292	255	355	316	245	312	215	385	223	273	229	320
3	312	237	286	246	350	309	236	307	188	377	199	261	216	316
5	310	233	282	245	337	305	229	303	182	370	196	258	214	314
10	302	230	278	239	320	293	224	293	180	351	192	253	212	298
15	287	229	275	237	290	284	222	277	177	326	191	249	212	275
25	254	221	267	230	248	249	214	236	176	273	190	242	209	237
30	239	216	260	229	230	231	208	221	176	250	190	239	209	222
40	221	196	240	225	211	211	191	197	175	232	189	229	206	201
50	204	181	225	209	193	199	177	188	174	216	188	217	200	185
100	155	139	176	153	163	162	134	153	165	163	177	161	166	146

TPM value	S99	S100	S101	S102	S103	S104	S105	S106	S107	S108	S109	S110	S111	S112
0	2588	2588	2588	2588	2588	2588	2588	2588	2588	2588	2588	2588	2588	2588
1	186	203	362	268	231	228	251	245	299	189	194	178	321	210
3	164	203	351	245	215	159	181	239	291	145	176	145	312	203
5	158	150	349	243	212	145	163	238	289	134	165	139	309	203
10	156	127	340	238	208	136	159	237	286	124	159	134	296	198
15	156	113	313	236	205	133	159	234	270	123	158	134	277	193
25	155	97	269	233	202	130	159	225	242	119	154	134	238	190
30	154	93	254	233	201	127	158	219	225	119	152	132	226	189
40	154	84	225	230	200	126	157	209	202	119	151	131	193	187
50	151	82	210	226	197	125	156	201	178	119	150	131	180	182
100	141	68	162	213	182	122	152	160	138	115	133	123	137	149

TPM value	S113	S114	S115	S116	S117	S118	S119	S120	S121	S122	S123	S124	S125
0	2588	2588	2588	2588	2588	2588	2588	2588	2588	2588	2588	2588	2588
1	364	290	263	301	477	275	283	318	321	309	205	210	209
3	353	280	253	296	446	270	278	314	315	300	194	144	180
5	348	275	249	292	405	266	274	311	312	299	192	125	174
10	334	271	246	281	326	259	267	294	282	287	188	106	168
15	302	261	245	257	298	251	259	282	246	265	186	100	167
25	264	235	236	233	252	221	232	255	212	232	184	93	166
30	244	225	226	211	233	206	220	244	198	227	184	90	165
40	220	207	207	193	207	190	202	218	176	212	183	89	165
50	205	184	192	186	195	177	185	195	162	198	179	89	165
100	155	143	152	141	154	139	144	153	130	151	152	86	157

Files for genome browsers

Various different genome browsers can be used for viewing the aligned count data in genomic context with a variety of different annotations. Figure 7 shows an example of the data viewed with the IGV Genome Browser available at the Broad Institute.

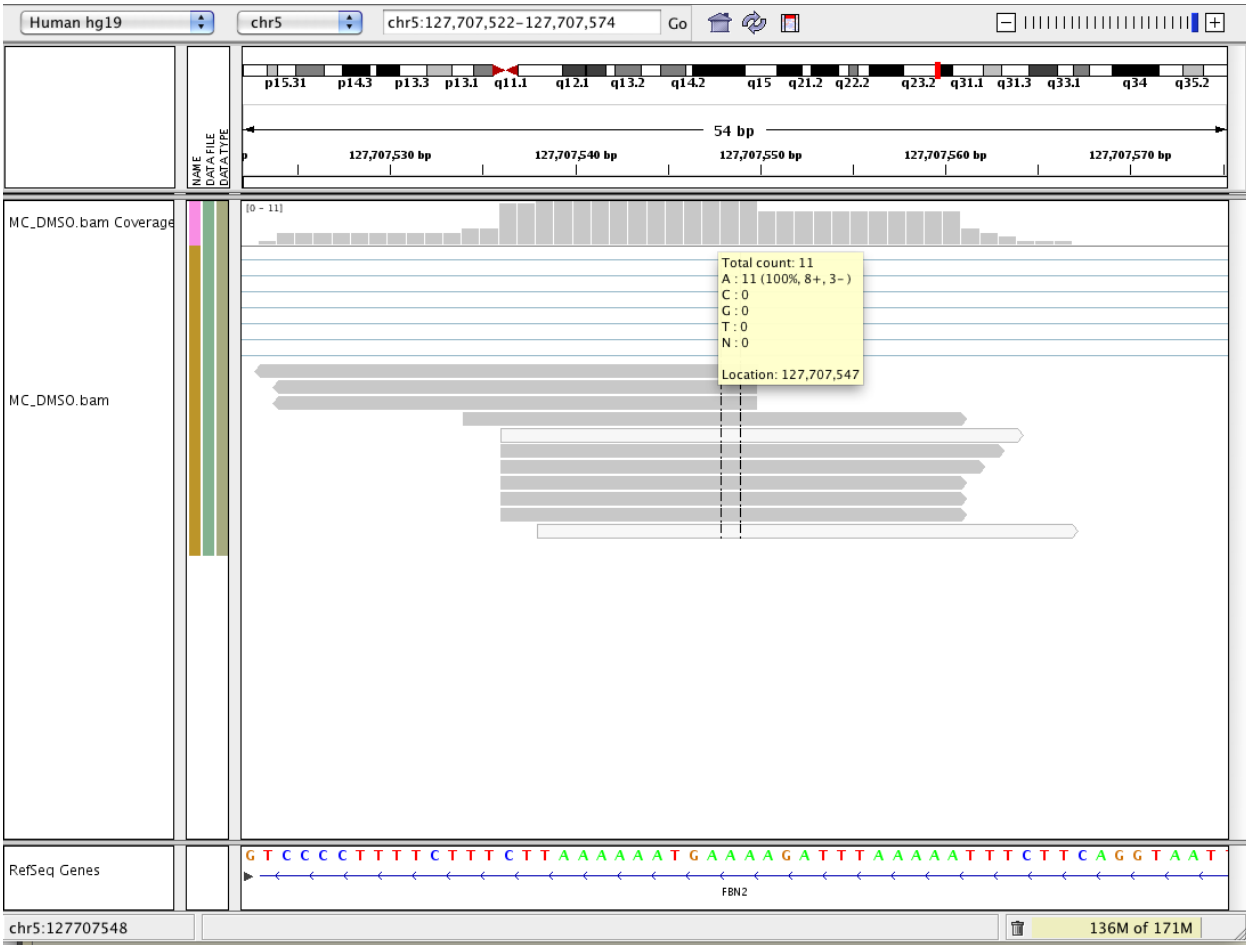

Figure S7: Snapshot from IGV Genome Browser.

Quality control

Quality control is an important step in the analysis to assess the overall quality of the samples, to see how well the replicates correlate with each other and to identify possible outliers. Here, several widely used methods have been used for quality control.

Expression values

Figure S8 visualizes the expression value distribution across the sample set and the table shows the minimum, median, mean and maximum expression values of the normalized samples.

	x
Min.	0
Median	0
Mean	819
Max.	199384

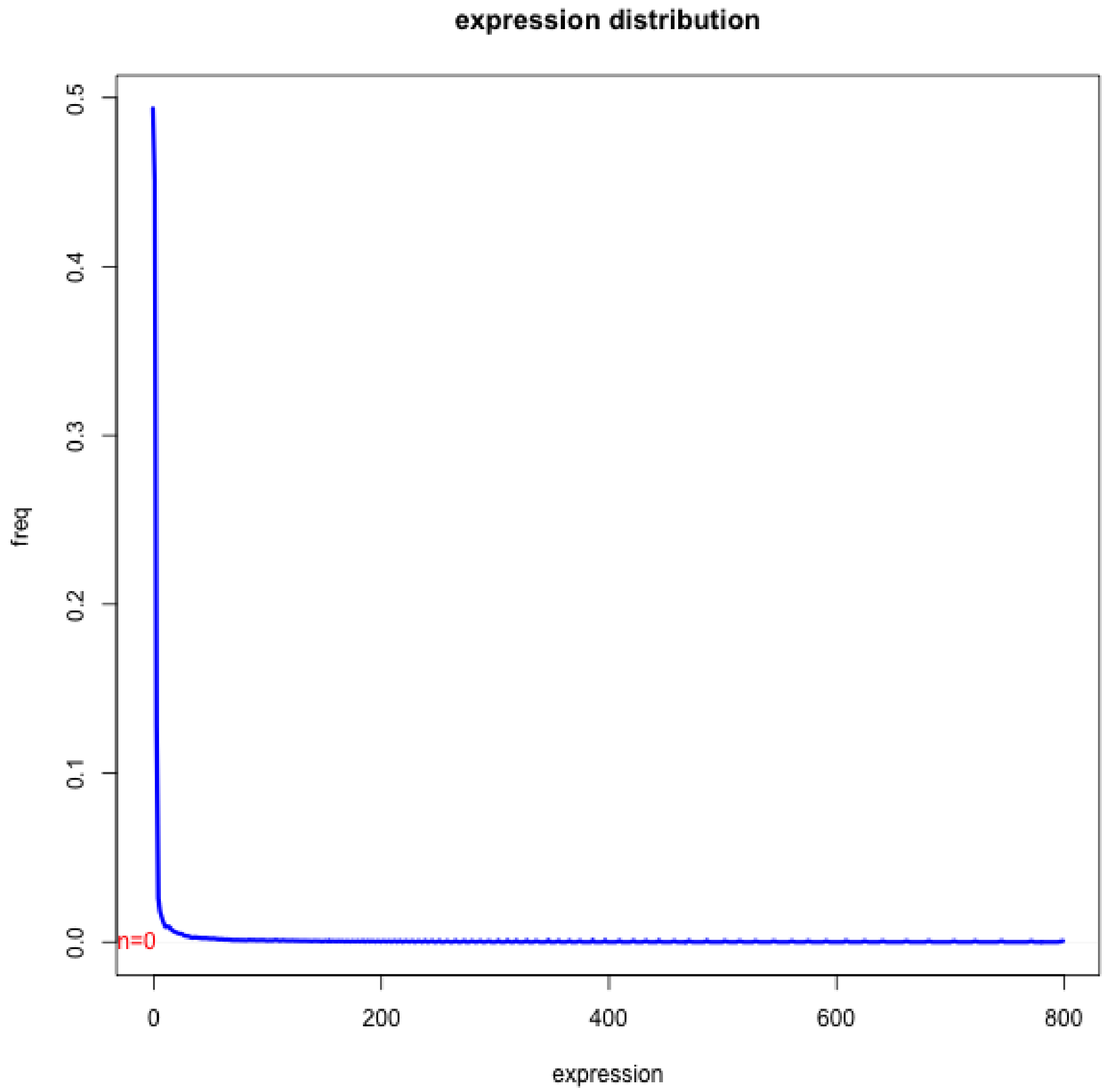

Figure S8: A curve describing a part of the expression value distribution of the samples in this study. Most genes yield very small expression values and only a few genes have high values.

Correlations

Between sample correlation values describe the similarity between the samples in a general level, when all measurement features of all samples are taken into consideration. In this analysis the so called *Spearman's metrics* is used which describes the between sample similarity on a scale of 0-1. Value 0 means perfect uncorrelation between the samples whereas value 1 means perfect correlation between them.

The correlation values between all possible pairs of samples are visualized for both spike-in reads and genomic reads in the figure below.

Table S5: GroupWise correlation values for spike-ins.

GroupName	minCor	meanCor	medianCor	maxCor	corSD
spike-in	0.596	0.889	0.902	0.995	0.065

Table S6: GroupWise correlation values for genomic reads.

GroupName	minCor	meanCor	medianCor	maxCor	corSD
tissue	0.754	0.812	0.818	0.853	0.03
NSCLC	0.546	0.686	0.69	0.796	0.041
Control	0.641	0.731	0.732	0.804	0.02

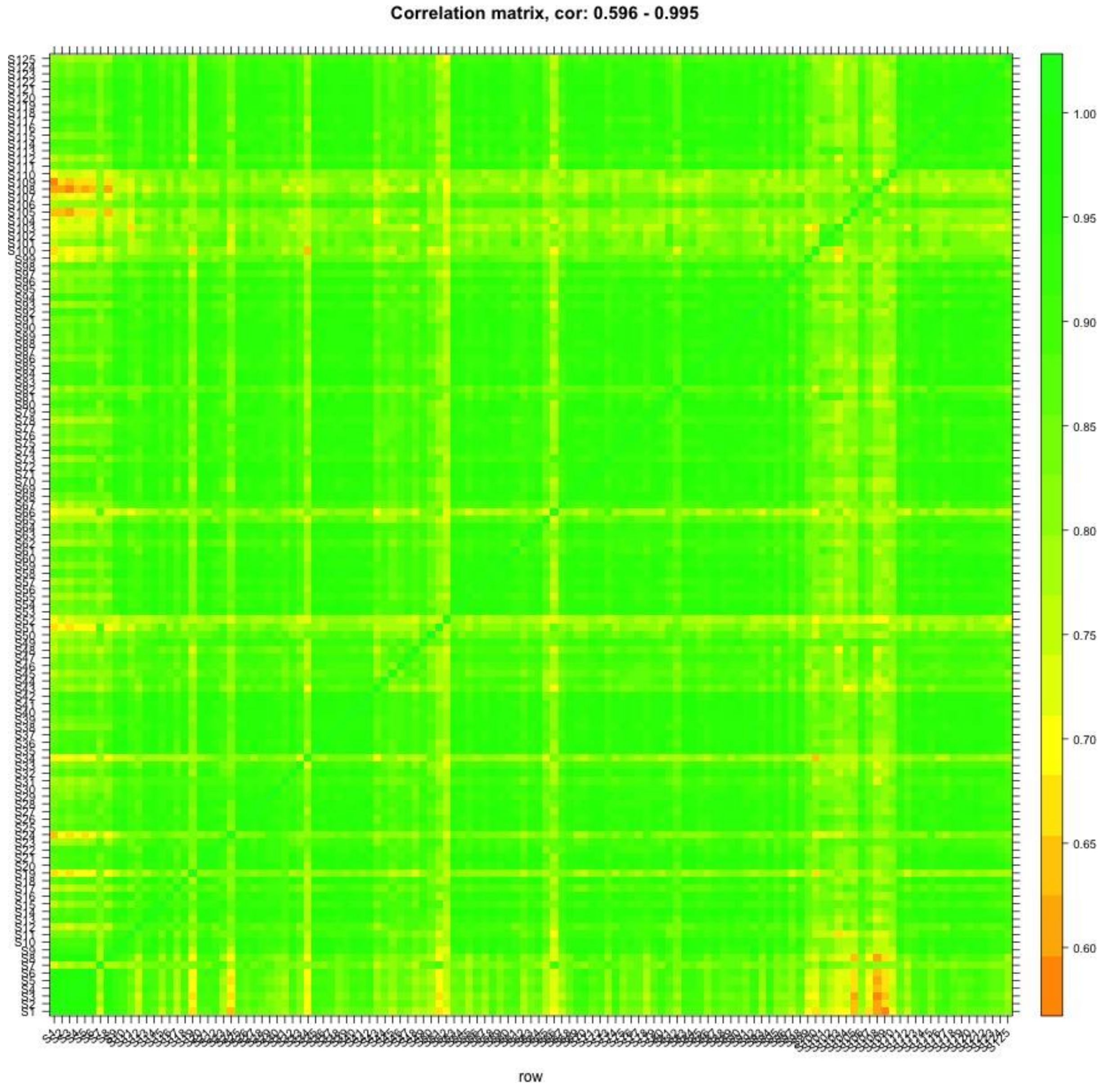

Figure S9: Sample correlations for spike-in reads.

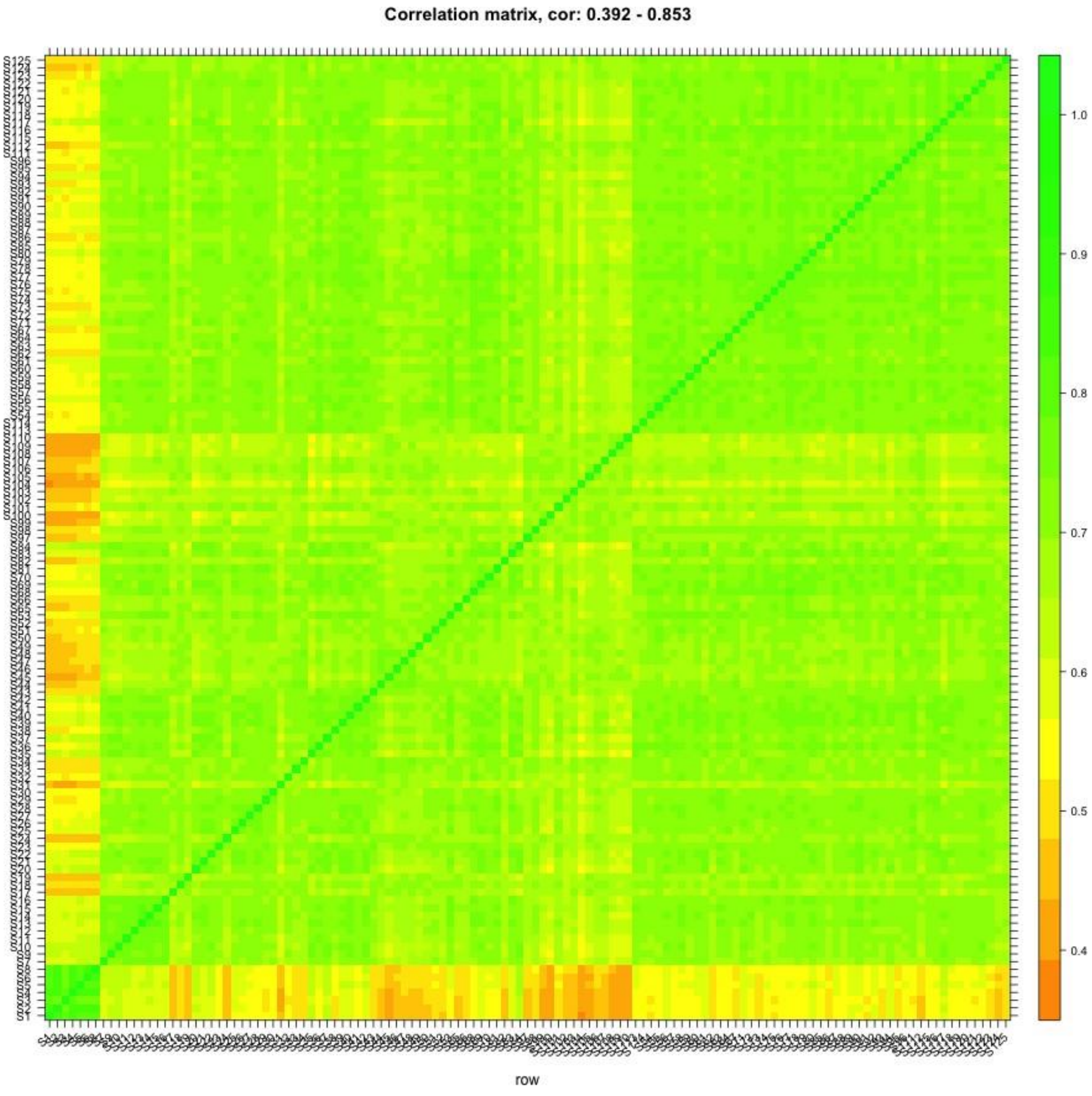

Figure S10: Sample correlations for genomic reads.

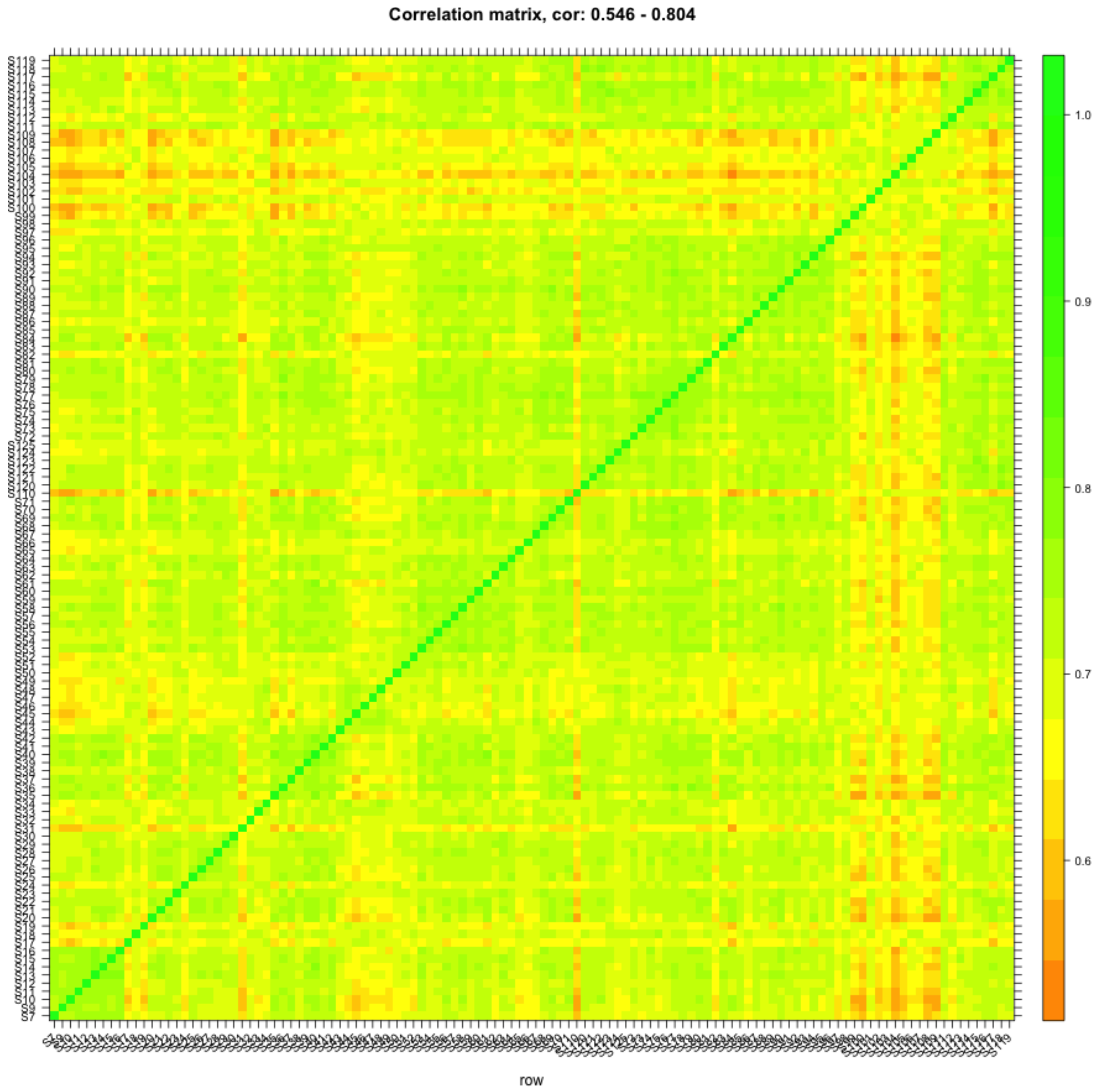

Figure S11: Sample correlations for genomic reads without tissue samples.

Hierarchical clustering

In hierarchical clustering the samples are grouped according to their general similarity when all the mea- surements of all the samples are taken into consideration. In this analysis the samples were clustered with Euclidean metrics.

The result of the cluster analysis can be visualised as a *dendrogram*, which is an out branching graph where the most similar samples (in another words best correlating) can be found in the branches that are nearest to one another.

Dendrograms produced by cluster analysis for both spike-in reads and genomic reads are shown in the figures below.

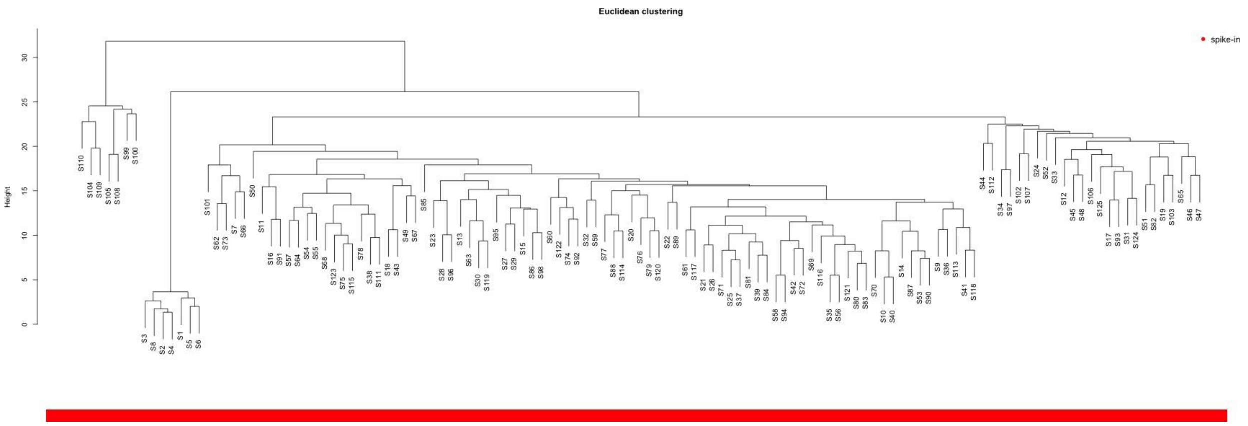

Figure S12: Hierarchical clustering for spike-in reads

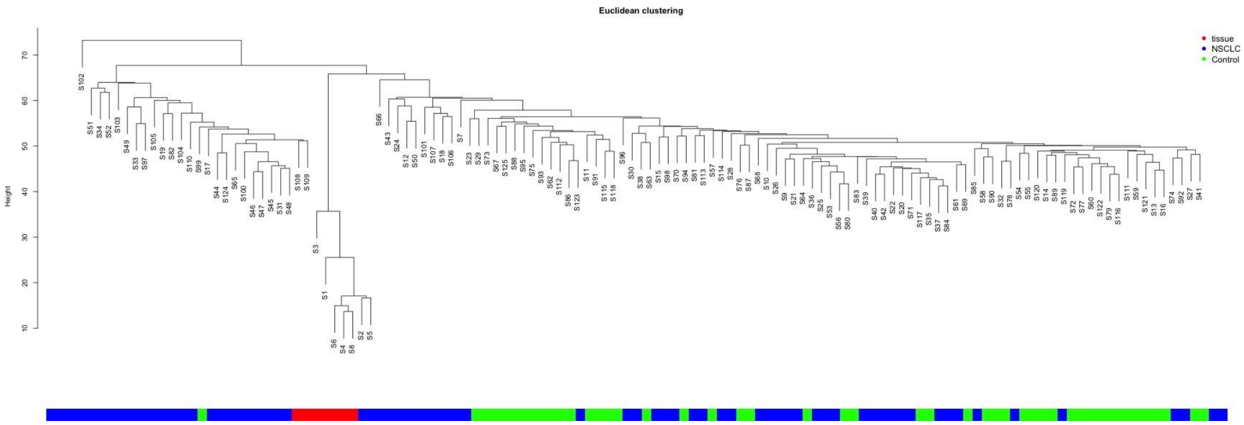

Figure S13: Hierarchical clustering for genomic reads

PCA

The sample relations can also be studied by the means of a Principal Component Analysis (PCA) which is an ordination technique complementary to clustering. Ordination orders objects so that similar objects are placed near each other and dissimilar objects are placed further from each other.

In PCA analysis the sample relationships can be visualized in three dimensional space. Figures below show the PCA plots for both spike-in reads and genomic reads.

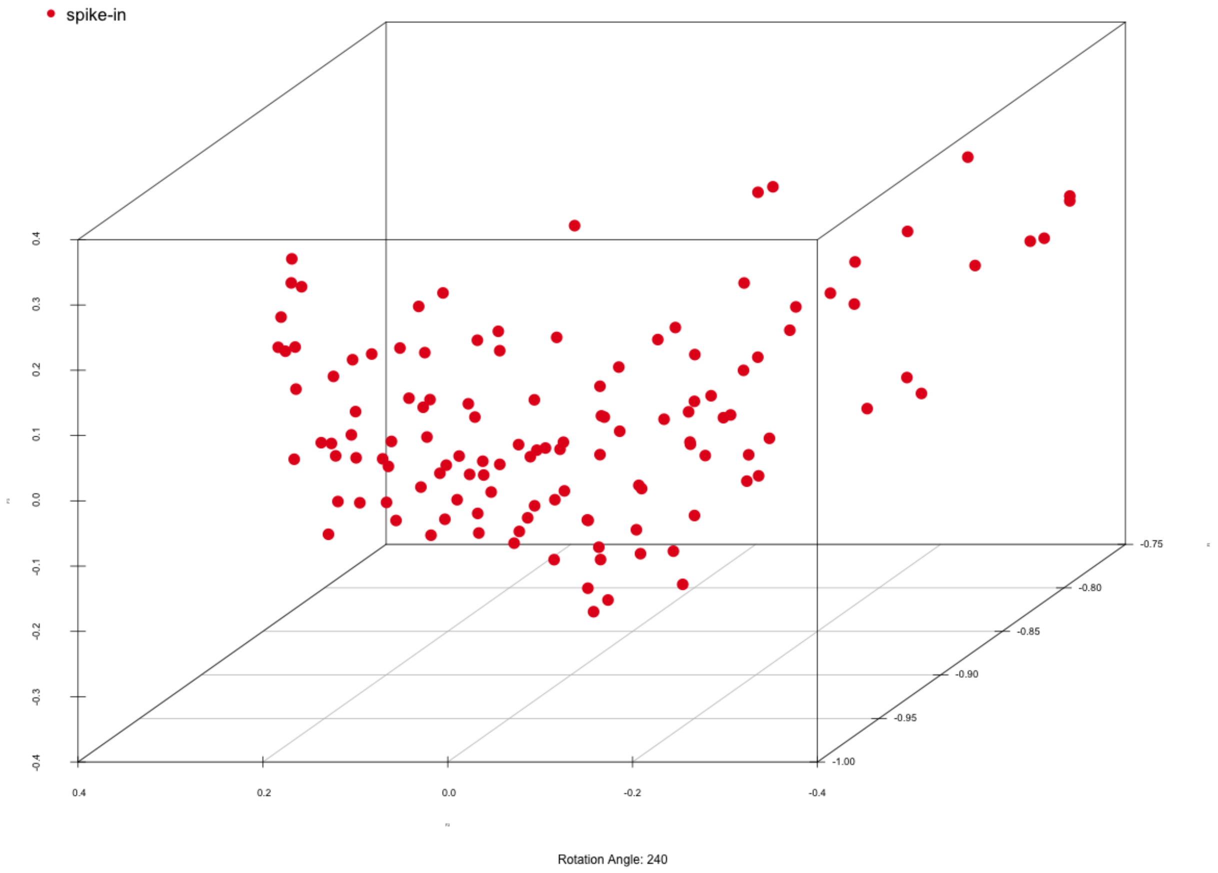

Figure S14: PCA plot for spike-in reads

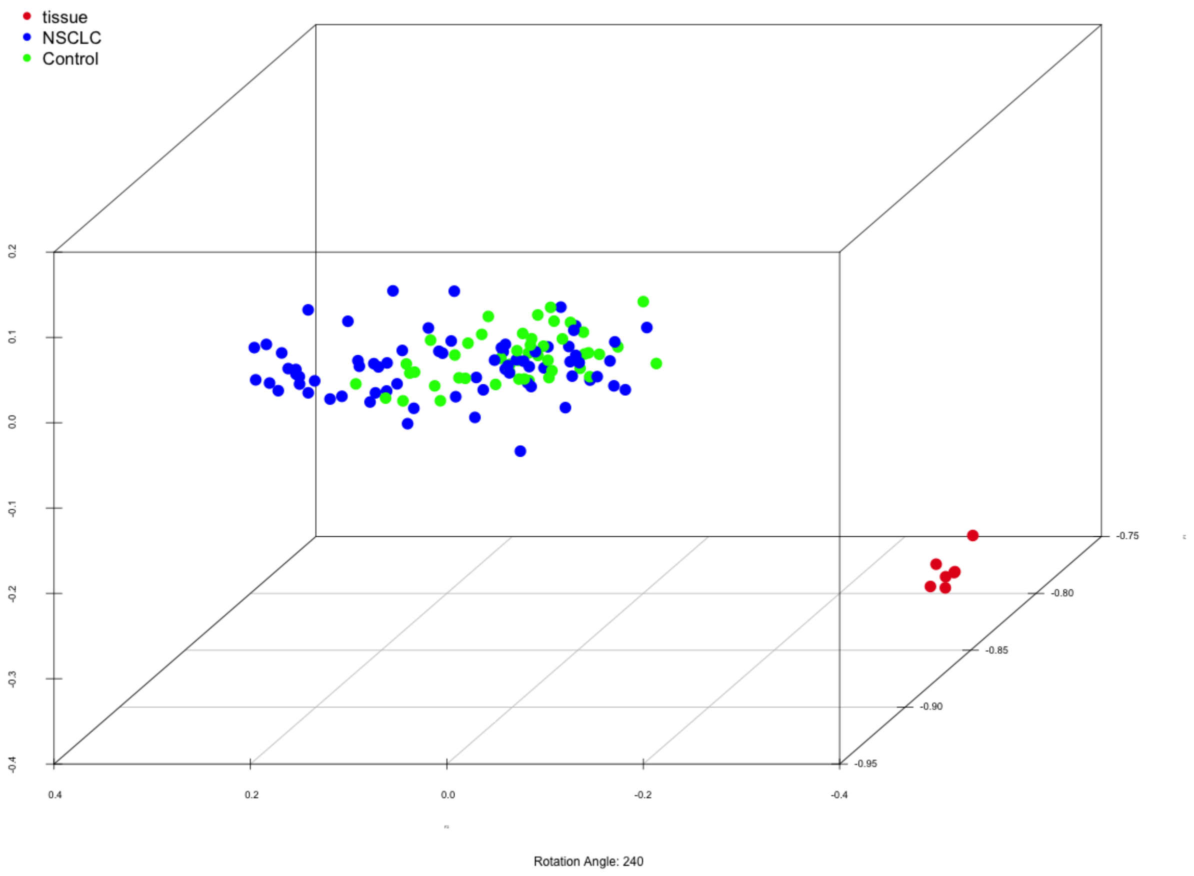

Figure S15: PCA plot for genomic read

Differential expression analysis

The following comparisons were performed to detect differentially expressed genes between groups:

x
NSCLC_vs_control

R package limma was used to perform the statistical testing. More information on the package can also be found in the limma user guide.

Filtering parameters

When filtering up- and down-regulated (i.e. differentially expressed = DE) genes between certain conditions (groups) fold changes and p-values (or multiple testing corrected p-values) calculated during statistical testing are used as filtering criteria.

All of the measured genes are filtered to list those that show the strongest evidence for being differentially expressed between the compared groups.

Short descriptions of fold change and p-values:

- **Fold change** (FC) describes the size of the difference in gene expression between the compared groups. In this analysis it results from linear modeling process performed with Limma package. Fold changes are often expressed as log2-transformed, where value 0 means ‘no change’ and 1 means doubled value and -1 means halved value. The values are always in relation to the group used as a base level group (reference).
- **P-value** describes the reliability of the change in expression value between the compared groups. Better (i.e. smaller) p-value is given for those genes that show homogeneous behaviour inside each group and yet clearly differ between the compared groups. In this analysis the p-values used for filtering can be either so called modified t-test p-values or *FDR* (false-discovery-rate) p-values which are both produced by Limma. Modified t-test p-values are not corrected for multiple testing. FDR p-values are used to control the rate of false positive findings in the result list and have been generally found to perform better than traditional p-values.

Choosing thresholds for filtering

The choice of the thresholds for p-value and fold change used for filtering the differentially expressed (DE) genes is not a trivial task. There is no one correct way or method to determine the thresholds but the choice is based on different aspects of each study. Different thresholds can also be used for filtering the data for different purposes. For example, often very strict thresholds are chosen when the data is filtered to be included in a publication. Then the result list will contain very few false positive findings but on the other hand many true positives are left outside the result set. Because of this it is typically useful to use less stringent thresholds for filtering data for internal research purposes or functional analysis when a larger proportion of possible false positive findings can be tolerated. Cluster analysis of the filtered genes can also be used as a means for choosing the filtering thresholds: such thresholds should be chosen, that the samples are grouping according to the known sample groups in the cluster analysis of the filtered genes.
