# Supplementary material for: Serum Insights: Leveraging the Power of miRNA Profiling as an Early Diagnostic Tool for Non-Small Cell Lung Cancer
Source: Cancers (Basel). 2023 Oct 10;15(20):4910. doi: 10.3390/cancers15204910 (PMC10605272; doi:10.3390/cancers15204910)
Supplement: Supplementary file 1 [file cancers-15-04910-s001.zip › Tables S9¿CS14.pdf]

Table S9. DEmiRNAs between cancerous and non-cancerous patients

Subgroups	upregulated DEmiRNAs	downregulated DEmiRNAs
NSCLC vs COPD	27	0
NSCLC vs noCOPD	12	0
COPD vs noCOPD	6	13
AC vs COPD	892	12
AC vs noCOPD	896	13
AC vs SCC	425	0
SCC vs COPD	15	0
SCC vs noCOPD	12	0

Table S10. Shared DE miRNAs between subgroups

ID	NSCLC_vs_COPD	NSCLC_vs_noCOPD	COPD_vs_noCOPD
hsa-let-7e-3p	1	0	0
hsa-let-7i-5p	0	0	1
hsa-miR-1225-5p	1	0	0
hsa-miR-135a-5p	1	0	0
hsa-miR-135b-5p	1	1	0
hsa-miR-143-5p	1	0	0
hsa-miR-181c-5p	0	0	-1
hsa-miR-190b	1	0	0
hsa-miR-191-3p	0	0	1
hsa-miR-194-3p	1	0	-1
hsa-miR-200a-5p	1	1	0
hsa-miR-202-3p	0	1	1
hsa-miR-205-5p	0	1	0
hsa-miR-23b-5p	0	0	-1
hsa-miR-29b-1-5p	1	0	-1
hsa-miR-3173-3p	1	1	0
hsa-miR-3178	0	1	0
hsa-miR-3180	1	0	0
hsa-miR-3180-3p	1	0	0
hsa-miR-3199	0	0	1
hsa-miR-4488	1	0	0
hsa-miR-4492	0	1	0
hsa-miR-4532	1	0	0
hsa-miR-454-3p	0	0	-1
hsa-miR-4637	0	0	-1
hsa-miR-4646-5p	0	1	0
hsa-miR-4723-3p	0	0	1
hsa-miR-485-3p	0	0	-1
hsa-miR-485-5p	0	0	-1
hsa-miR-491-5p	1	0	0
hsa-miR-511-3p	0	0	-1

hsa-miR-624-5p	0	0	-1
hsa-miR-6763-5p	0	0	-1
hsa-miR-6772-3p	1	0	0
hsa-miR-6809-5p	0	1	0
hsa-miR-6819-3p	1	1	0
hsa-miR-6821-5p	1	1	0
hsa-miR-6828-3p	1	0	0
hsa-miR-6837-3p	1	0	0
hsa-miR-6852-3p	0	0	1
hsa-miR-6876-5p	1	0	0
hsa-miR-6891-5p	1	0	0
hsa-miR-766-5p	1	0	0
hsa-miR-7704	1	0	-1
hsa-miR-8072	1	0	0
hsa-miR-873-3p	1	0	0
hsa-miR-92a-1-5p	1	0	0
hsa-miR-96-5p	0	0	-1

Table S11. NSCLC vs control_GO_BP_results

GO Category	p-value	No of genes	No of miRNAs
cellular nitrogen compound metabolic process	4.23E-132	971	14
gene expression	4.60E-101	229	13
biosynthetic process	5.66E-93	806	15
viral process	1.60E-58	159	14
symbiosis. encompassing mutualism through parasitism	5.42E-58	171	14
cellular protein modification process	6.23E-51	463	15
biological process	2.92E-44	2423	16
catabolic process	6.18E-43	393	14
small molecule metabolic process	2.38E-39	433	14
response to stress	3.06E-37	438	14
mitotic cell cycle	1.12E-35	116	13
cellular protein metabolic process	2.43E-35	126	11
membrane organization	1.99E-33	155	13
neurotrophin TRK receptor signaling pathway	1.52E-31	80	12
cellular component assembly	1.96E-31	270	15
cell death	2.71E-31	214	14
macromolecular complex assembly	3.08E-31	203	15
nucleobase-containing compound catabolic process	3.31E-29	200	13
mRNA metabolic process	1.05E-25	70	12
RNA metabolic process	4.41E-24	76	12
DNA metabolic process	3.75E-21	169	14
Fc-epsilon receptor signaling pathway	1.58E-19	48	13
protein complex assembly	1.15E-18	159	15
blood coagulation	1.10E-17	98	13
activation of signaling protein activity involved in unfolded protein response	4.89E-17	30	10

TRIF-dependent toll-like receptor signaling pathway	4.89E-17	30	11
MyD88-independent toll-like receptor signaling pathway	2.47E-15	30	11
transcription initiation from RNA polymerase II promoter	1.55E-14	63	12
toll-like receptor 3 signaling pathway	2.05E-14	31	11
toll-like receptor 10 signaling pathway	5.53E-14	25	10
toll-like receptor TLR1:TLR2 signaling pathway	3.83E-13	25	10
toll-like receptor TLR6:TLR2 signaling pathway	3.83E-13	25	10
regulation of ubiquitin-protein ligase activity involved in mitotic cell cycle	4.62E-13	28	9
viral life cycle	6.04E-13	35	12
epidermal growth factor receptor signaling pathway	6.04E-13	54	12
immune system process	1.19E-12	263	14
positive regulation of ubiquitin-protein ligase activity involved in mitotic cell cycle	1.35E-12	27	9
toll-like receptor 5 signaling pathway	1.98E-12	25	10
toll-like receptor 9 signaling pathway	3.07E-12	26	10
transcription. DNA-templated	9.34E-12	407	14
G2/M transition of mitotic cell cycle	9.34E-12	44	12
fibroblast growth factor receptor signaling pathway	1.13E-11	51	13
toll-like receptor 4 signaling pathway	2.84E-11	31	11
anaphase-promoting complex-dependent proteasomal ubiquitin-dependent protein catabolic process	4.37E-11	30	9
generation of precursor metabolites and energy	4.50E-11	73	14
mitotic nuclear envelope disassembly	6.10E-11	17	10
toll-like receptor 2 signaling pathway	1.07E-10	26	10
stress-activated MAPK cascade	1.11E-10	22	10
toll-like receptor signaling pathway	1.92E-10	33	11
RNA splicing	2.80E-10	78	13
hexose transport	6.30E-10	17	10
DNA damage response. signal transduction by p53 class mediator resulting in cell cycle arrest	8.72E-10	26	10
negative regulation of ubiquitin-protein ligase activity involved in mitotic cell cycle	1.09E-09	23	8
Fc-gamma receptor signaling pathway involved in phagocytosis	2.00E-09	22	10
G1/S transition of mitotic cell cycle	2.50E-09	48	11
mRNA processing	3.78E-09	110	13
endoplasmic reticulum unfolded protein response	4.15E-09	39	11
post-translational protein modification	8.33E-09	36	9
MyD88-dependent toll-like receptor signaling pathway	9.22E-09	28	10
positive regulation of protein insertion into mitochondrial membrane involved in apoptotic signaling pathway	2.89E-08	14	9
ribonucleoprotein complex assembly	3.19E-08	39	11
mRNA splicing. via spliceosome	3.85E-08	60	12
platelet activation	4.03E-08	43	12
regulation of glucose transport	4.22E-08	14	10

cellular component disassembly involved in execution phase of apoptosis	5.50E-08	16	8
termination of RNA polymerase II transcription	5.99E-08	17	11
cellular component movement	6.46E-08	31	11
protein N-linked glycosylation via asparagine	7.69E-08	27	10
innate immune response	9.15E-08	123	13
SRP-dependent cotranslational protein targeting to membrane	1.23E-07	32	10
chromatin organization	1.52E-07	33	11
phosphatidylinositol-mediated signaling	1.82E-07	34	12
negative regulation of apoptotic process	2.12E-07	139	13
cell cycle	3.45E-07	163	14
cell junction organization	3.72E-07	36	12
intrinsic apoptotic signaling pathway	4.87E-07	22	11
mRNA 3'-end processing	5.60E-07	15	9
viral transcription	9.60E-07	21	9
regulation of cellular amino acid metabolic process	1.00E-06	17	7
nucleotide-binding domain. leucine rich repeat containing receptor signaling pathway	1.31E-06	14	10
vesicle-mediated transport	1.33E-06	167	15
antigen processing and presentation of exogenous peptide antigen via MHC class II	1.45E-06	30	11
intracellular transport of virus	4.27E-06	10	8
nuclear-transcribed mRNA catabolic process. nonsense-mediated decay	4.52E-06	32	11
protein targeting	4.54E-06	57	11
transcription from RNA polymerase II promoter	4.88E-06	113	14
cellular lipid metabolic process	5.43E-06	29	10
post-Golgi vesicle-mediated transport	7.15E-06	16	9
regulation of transcription from RNA polymerase II promoter in response to hypoxia	9.13E-06	12	8
transforming growth factor beta receptor signaling pathway	9.66E-06	43	11
translational termination	1.60E-05	23	9
energy reserve metabolic process	1.60E-05	23	10
nucleobase-containing small molecule metabolic process	1.68E-05	17	10
positive regulation of type I interferon production	1.98E-05	18	11
protein polyubiquitination	2.95E-05	38	11
nuclear-transcribed mRNA catabolic process. deadenylation-dependent decay	3.95E-05	17	9
apoptotic signaling pathway	4.18E-05	30	12
cytoskeleton organization	4.63E-05	109	12
nucleotide-binding oligomerization domain containing signaling pathway	5.96E-05	9	8
platelet degranulation	8.56E-05	17	10
antigen processing and presentation of exogenous peptide antigen via MHC class I	9.09E-05	23	9
antigen processing and presentation of exogenous peptide antigen via MHC class I. TAP-dependent	9.87E-05	22	9

axon guidance	0.000182637	78	12
telomere maintenance via semi-conservative replication	0.000192375	8	5
glucose transport	0.000203894	20	10
extracellular matrix disassembly	0.000209369	22	10
protein maturation	0.000210182	39	13
in utero embryonic development	0.00021689	67	12
DNA strand elongation involved in DNA replication	0.000275501	10	6
translational initiation	0.000275501	46	10
purine nucleobase metabolic process	0.00029211	13	6
androgen receptor signaling pathway	0.000373334	17	11
apoptotic process	0.000451311	147	13
positive regulation of viral transcription	0.000486799	13	8
regulation of interferon-gamma-mediated signaling pathway	0.000511582	8	7
regulation of type I interferon-mediated signaling pathway	0.000659757	9	7
leukocyte migration	0.000798242	23	11
negative regulation of transcription from RNA polymerase II promoter	0.000798242	168	14
DNA damage response. detection of DNA damage	0.000841576	7	6
cytokine-mediated signaling pathway	0.000867079	54	13
nitric oxide metabolic process	0.000909585	8	6
transcription elongation from RNA polymerase II promoter	0.001062741	18	8
insulin receptor signaling pathway	0.001131027	32	11
water-soluble vitamin metabolic process	0.001394703	14	7
phosphatidylinositol biosynthetic process	0.001521324	15	10
telomere maintenance via recombination	0.001625016	9	5
nuclear-transcribed mRNA poly(A) tail shortening	0.002192814	11	7
cellular response to hypoxia	0.002459043	32	12
negative regulation of intrinsic apoptotic signaling pathway	0.002941436	14	7
long-chain fatty-acyl-CoA biosynthetic process	0.003816109	6	4
anatomical structure morphogenesis	0.004028966	21	10
homeostatic process	0.004203422	115	11
antigen processing and presentation of peptide antigen via MHC class I	0.005009354	29	10
negative regulation of type I interferon production	0.00508742	11	10
vitamin metabolic process	0.005139884	14	7
histone mRNA metabolic process	0.006009072	5	6
positive regulation of muscle cell differentiation	0.006198935	9	9
response to virus	0.006714322	35	10
mRNA transport	0.006764467	22	12
cofactor metabolic process	0.006834602	39	12
positive regulation of apoptotic process	0.007513852	77	13
viral protein processing	0.007934651	6	7
Notch signaling pathway	0.00846619	37	11
nucleotide-excision repair. DNA gap filling	0.008508258	8	6
cell motility	0.008617324	81	13
nucleotide-excision repair. DNA damage removal	0.009236484	7	7

DNA biosynthetic process	0.009236484	12	7
cellular response to glucose starvation	0.010061119	10	5
cell junction assembly	0.010103868	12	8
glucose metabolic process	0.010103868	29	11
regulation of apoptotic process	0.010103868	50	12
RNA splicing. via transesterification reactions	0.010967564	9	7
nucleocytoplasmic transport	0.010967564	56	13
membrane fusion	0.012160342	11	9
negative regulation of anoikis	0.012721231	10	7
transport	0.012727424	535	15
regulation of nitric-oxide synthase activity	0.013212524	7	6
DNA repair	0.013212524	83	13
ATP-dependent chromatin remodeling	0.013244739	13	11
transcription-coupled nucleotide-excision repair	0.013394266	12	7
respiratory electron transport chain	0.013394266	19	11
modulation by virus of host morphology or physiology	0.013480131	6	7
COPII vesicle coating	0.015657473	7	4
positive regulation of transcription. DNA-templated	0.017960676	139	14
carbohydrate metabolic process	0.018245417	138	15
osteoblast differentiation	0.018626649	34	12
endosomal transport	0.019706852	18	11
response to amino acid	0.020604471	13	7
negative regulation of extrinsic apoptotic signaling pathway	0.020604471	17	8
type I interferon signaling pathway	0.02063763	14	8
cell proliferation	0.021292294	95	13
regulation of cell cycle	0.022342712	45	13
negative regulation of neuron apoptotic process	0.023213538	41	12
regulation of insulin secretion	0.024452404	20	9
negative regulation of Schwann cell proliferation	0.024775925	4	7
translational elongation	0.026088388	26	9
positive regulation of axonogenesis	0.026959213	10	7
response to unfolded protein	0.029868151	16	7
phospholipid metabolic process	0.029868151	26	12
regulation of microtubule-based process	0.030388944	8	9
cytoskeleton-dependent intracellular transport	0.031012709	19	9
L-ascorbic acid metabolic process	0.031179014	5	4
cotranslational protein targeting to membrane	0.031179014	5	8
mRNA splice site selection	0.032555936	10	8
plus-end-directed vesicle transport along microtubule	0.033692119	4	4
glycerophospholipid biosynthetic process	0.034115364	14	8
release of cytochrome c from mitochondria	0.034279379	11	7
protein localization to endosome	0.034682881	6	5
NLS-bearing protein import into nucleus	0.038182768	8	5
cellular response to nerve growth factor stimulus	0.043628312	8	5
response to calcium ion	0.045888037	20	8
peptidyl-threonine phosphorylation	0.045888037	16	9
cellular response to amino acid stimulus	0.047918665	17	8

Table S12. NSCLC vs control_GO_CC_results

GO Category	p-value	No of genes	No of miRNAs
organelle	6.50E-269	1890	15
nucleoplasm	2.07E-70	338	13
protein complex	9.83E-64	749	15
cytosol	1.30E-56	574	15
cellular component	1.75E-49	2490	16
focal adhesion	4.33E-07	117	13
microtubule organizing center	5.69E-07	87	11
nuclear chromatin	0.005362329	49	13
cytoplasmic stress granule	0.009041759	17	8
nucleolus	0.013837147	158	14
lysosomal lumen	0.017379689	13	7
heterochromatin	0.035105542	16	9
coated pit	0.046519624	20	10
extracellular vesicular exosome	0.046519624	525	15

Table S13. NSCLC vs control_GO_MF_results

GO Category	p-value	No of genes	No of miRNAs
ion binding	7.11E-65	995	15
molecular function	2.91E-57	2486	16
RNA binding	1.33E-50	427	15
enzyme binding	8.85E-45	302	15
protein binding transcription factor activity	6.63E-29	130	14
poly(A) RNA binding	1.49E-25	353	15
nucleic acid binding transcription factor activity	1.77E-14	177	14
cytoskeletal protein binding	9.05E-13	145	13
translation factor activity. nucleic acid binding	3.43E-08	35	10
transcription factor binding	3.43E-08	124	14
transcription coactivator activity	6.58E-06	72	13
enzyme regulator activity	6.58E-06	130	14
small conjugating protein binding	0.000304443	23	11
transcription corepressor activity	0.000689978	53	14
chromatin binding	0.021834032	114	14
androgen receptor binding	0.023467259	15	12
ligase activity	0.026172248	97	14
nuclear localization sequence binding	0.032175028	6	4

Table S14. NSCLC vs control_KEGG_results

KEGG pathway	p-value	No of genes	No of miRNAs
Fatty acid biosynthesis	4.74E-09	2	3
Adherens junction	2.23E-06	29	12

p53 signaling pathway	2.23E-06	34	13
Oocyte meiosis	6.46E-06	39	10
Cell cycle	1.25E-05	46	11
Central carbon metabolism in cancer	1.25E-05	27	11
Protein processing in endoplasmic reticulum	1.25E-05	60	12
Hippo signaling pathway	1.73E-05	46	14
Viral carcinogenesis	1.87E-05	59	12
Lysine degradation	0.000205089	16	10
Shigellosis	0.000208027	27	11
Small cell lung cancer	0.000209101	34	10
Hepatitis B	0.000209101	46	11
Proteoglycans in cancer	0.000256847	58	13
Bacterial invasion of epithelial cells	0.000629281	30	12
Prostate cancer	0.000683627	33	11
Spliceosome	0.000806205	42	12
Pancreatic cancer	0.000941618	24	11
Colorectal cancer	0.001026565	23	11
Glioma	0.001052948	23	10
RNA transport	0.001052948	52	12
Chronic myeloid leukemia	0.001170238	26	11
Endometrial cancer	0.002560307	20	10
Bladder cancer	0.002909431	17	8
HTLV-I infection	0.002909431	75	14
ErbB signaling pathway	0.003473008	29	12
Focal adhesion	0.003473008	62	13
Neurotrophin signaling pathway	0.003549326	40	12
Base excision repair	0.003713923	12	7
Fatty acid metabolism	0.004813961	8	5
Transcriptional misregulation in cancer	0.008007297	43	11
TGF-beta signaling pathway	0.009467253	25	12
HIF-1 signaling pathway	0.009591052	34	12
Sphingolipid signaling pathway	0.009808177	35	12
FoxO signaling pathway	0.01289245	42	13
Thyroid cancer	0.014660204	12	8
Apoptosis	0.016905608	26	11
ECM-receptor interaction	0.017653143	20	8
Progesterone-mediated oocyte maturation	0.025719409	28	11
Renal cell carcinoma	0.029622936	21	11
DNA replication	0.038149677	13	7
TNF signaling pathway	0.038149677	30	11
Prolactin signaling pathway	0.038149677	20	13
Influenza A	0.038149677	49	13
Endocytosis	0.039461589	54	13
Thyroid hormone signaling pathway	0.043921423	34	12
Epstein-Barr virus infection	0.044483181	55	11
Nucleotide excision repair	0.045804484	16	8
